# Electrophysiological characterization of the hERG R56Q LQTS variant and targeted rescue by the activator RPR260243

**DOI:** 10.1085/jgp.202112923

**Published:** 2021-08-16

**Authors:** Jacob M. Kemp, Dominic G. Whittaker, Ravichandra Venkateshappa, ZhaoKai Pang, Raj Johal, Valentine Sergeev, Glen F. Tibbits, Gary R. Mirams, Thomas W. Claydon

**Affiliations:** 1 Department of Biomedical Physiology and Kinesiology, Simon Fraser University, Burnaby, Canada; 2 Centre for Mathematical Medicine & Biology, School of Mathematical Sciences, University of Nottingham, Nottingham, UK

## Abstract

Human Ether-à-go-go (hERG) channels contribute to cardiac repolarization, and inherited variants or drug block are associated with long QT syndrome type 2 (LQTS2) and arrhythmia. Therefore, hERG activator compounds present a therapeutic opportunity for targeted treatment of LQTS. However, a limiting concern is over-activation of hERG resurgent current during the action potential and abbreviated repolarization. Activators that slow deactivation gating (type I), such as RPR260243, may enhance repolarizing hERG current during the refractory period, thus ameliorating arrhythmogenicity with reduced early repolarization risk. Here, we show that, at physiological temperature, RPR260243 enhances hERG channel repolarizing currents conducted in the refractory period in response to premature depolarizations. This occurs with little effect on the resurgent hERG current during the action potential. The effects of RPR260243 were particularly evident in LQTS2-associated R56Q mutant channels, whereby RPR260243 restored WT-like repolarizing drive in the early refractory period and diastolic interval, combating attenuated protective currents. In silico kinetic modeling of channel gating predicted little effect of the R56Q mutation on hERG current conducted during the action potential and a reduced repolarizing protection against afterdepolarizations in the refractory period and diastolic interval, particularly at higher pacing rates. These simulations predicted partial rescue from the arrhythmic effects of R56Q by RPR260243 without risk of early repolarization. Our findings demonstrate that the pathogenicity of some hERG variants may result from reduced repolarizing protection during the refractory period and diastolic interval with limited effect on action potential duration, and that the hERG channel activator RPR260243 may provide targeted antiarrhythmic potential in these cases.

## Teaser

Kemp et al. show that the hERG channel activator RPR260243 restores the repolarizing current of a mutant variant of the channel associated with LQTS2 with little effect on its resurgent current. This drug represents a therapeutic opportunity for the treatment of arrhythmia associated with LQTS2.

## Introduction

Long QT syndrome (LQTS) is caused by dysfunction or altered regulation of one of several cardiac ion channel types and is characterized by a prolonged heart rate corrected QT (QT_c_) interval on the electrocardiogram in the absence of any obvious structural heart disease (Schwartz et al., 2012). With a risk of seizure, syncope, and sudden cardiac death associated with ventricular tachyarrhythmia, LQTS is a potentially fatal disorder with an acute onset. Approximately 25% of inherited LQTS cases and almost all cases of acquired LQTS arising from pharmacological QT_c_ prolongation are associated with the Kv11.1 cardiac voltage-gated K^+^ channel (also known as human Ether-à-go-go related gene; hERG), encoded by the *KCNH2* gene (Tester and Ackerman, 2014). hERG channels underlie the rapid component of the delayed rectifier current (*I*_Kr_) and are vital to the repolarization of cardiac tissue in phase 3 of the ventricular cardiac action potential (Sanguinetti et al., 1995; Trudeau et al., 1995). Loss of functional *I*_Kr_ current in inherited or acquired LQTS (designated LQTS type 2; LQTS2) results in delayed repolarization of cardiac muscle and prolongation of the cardiac action potential. Such delayed repolarization leaves cardiac tissue susceptible to early afterdepolarizations (EADs) and subsequent development of torsades de pointes and ventricular fibrillation (Curran et al., 1995; Sanguinetti et al., 1995, 1996; Smith et al., 1996; Lu et al., 2001).

The repolarizing drive arising from hERG channels in response to an action potential voltage waveform is readily apparent as a resurgent current and frequently used to characterize the functional consequences of inherited mutations or drug action. Less well studied is the repolarizing drive from hERG channels in response to premature depolarizations mimicking afterdepolarizations applied early in the refractory period (Miller, 1996; Lu et al., 2001; Perry et al., 2016). Robust transient repolarizing hERG “protective currents” arise because hERG channels are slow to deactivate (Lu et al., 2001; Du et al., 2010; Du et al., 2013). Depolarization drives hERG channels into open inactivated states from which they transition into a stable open relaxed state that involves stabilization of the activated voltage sensing region of the channel (Smith et al., 1996; Hardman et al., 2007; Al-Owais et al., 2009; Gustina and Trudeau, 2011, 2012; Ng et al., 2012; Cheng et al., 2013; Hull et al., 2014; Shi et al., 2019). Upon repolarization, channels are slow to close from this stable relaxed state, and this contributes to robust channel availability in the refractory period and thus the presence of protective currents in response to premature stimulations (Lu et al., 2001; Lu et al., 2003; Du et al., 2010; Du et al., 2013; McPate et al., 2009; Melgari et al., 2014; Ng et al., 2014; Perry et al., 2016). These studies demonstrate a potential critical role for hERG protective currents in resisting afterdepolarizations and suppressing arrhythmia generation.

There is significant interest in the therapeutic potential of small molecule activators of hERG channels, which may alleviate loss of channel function, in the treatment and management of LQTS. However, over-activation of hERG resurgent current during the action potential leading to abbreviated repolarization remains a significant concern. Some small molecules act as activators of hERG channels by slowing deactivation gating (known as type I activators), such as RPR260243; however, their potential influence on hERG protective currents has not been tested. It is plausible that sensitivity to risk of afterdepolarizations and triggered activity caused by LQTS2-associated mutations may be ameliorated by the action of RPR260243 without significant effect on hERG resurgent current during the action potential. Investigation of these effects would lead to improved understanding of the therapeutic potential of novel hERG activator compounds.

In this study, we combine electrophysiological characterization of hERG channel behavior at room and physiological temperatures with mathematical modeling of channel kinetics to demonstrate the therapeutic potential of activator compounds that slow hERG deactivation gating and enhance repolarization resistance to premature depolarizations. Our combined approach provides a thorough characterization of R56Q mutant channel kinetics and evaluates the role of reduced hERG protective currents caused by the fast deactivation gating phenotype of R56Q mutant channels, which are associated with sudden death, and predicts consequent risk of arrhythmogenicity. Furthermore, we investigate the ability of the activator compound, RPR260243, to enhance hERG protective currents and reduce afterdepolarization risk in WT and R56Q mutant channels. Our approach also aims to further validate a recently described kinetic modeling approach (Beattie et al., 2018; Lei et al., 2019a, b) by demonstrating faithful recapitulation and prediction of aberrant gating behavior in hERG channels at physiological temperature.

## Materials and methods

### Molecular biology

For expression in *Xenopus laevis* oocytes, hERG1a WT or R56Q mutant channel cDNA, subcloned into a pBluescript SKII expression vector, was linearized using *XbaI* to synthesize complementary RNA (cRNA) using the mMessage mMachine T7 Ultra cRNA Transcription Kit (Ambion). Mutations were generated by conventional overlap extension PCR using mutagenic primers synthesized by Sigma-Genosys and confirmed by sequencing (Eurofins MWG Operon). For expression in human embryonic kidney (HEK) cells, hERG1a WT or R56Q mutant channel cDNA was subcloned into a pcDNA3 vector.

### hERG channel expression in *Xenopus *oocytes

Experiments were conducted at 21°C in *Xenopus* oocytes and at 37°C in HEK cells. The latter approach enabled examination of the effects of the mutation at physiological temperature, and validation of the robustness and versatility of our mathematical modeling approach, which was previously used to recapitulate WT channel behavior at 21°C in Chinese hamster ovary cells using sinusoidal voltage protocols (Beattie et al., 2018) and Chinese hamster ovary cells at temperatures up to 37°C using a shortened step protocol (Lei et al., 2019a, b). Thus, characterization at 21°C and 37°C provided validation of mathematical modeling predictions of mutant behavior compared with that in WT, as well as validation of predictions using seed data sourced from complex recordings over long durations in *Xenopus* oocytes at 21°C and data from the shortened step protocol in HEK cells at 37°C. In agreement with the policies and procedures of the Simon Fraser University Animal Care Committee and the Canadian Council of Animal Care, stage V–VI oocytes were surgically isolated from *Xenopus* frogs (Nasco) and injected with hERG cRNA as described previously (Shi et al., 2019). The two-electrode voltage-clamp technique was used to record from injected oocytes with an OC-725C amplifier (Warner Instruments) and Digidata 1440 interface (Axon Instruments). Oocytes were perfused at 1 ml/min with ND96 solution containing (in mM) 96 NaCl, 3 KCl, 0.5 CaCl_2_, 1 MgCl_2_, and 5 HEPES, titrated to pH 7.4 with NaOH. Reagents were purchased from Sigma-Aldrich. Application of RPR260243 (Aobious) at different concentrations was achieved by 5 min perfusion of the compound at the desired concentration to allow complete exchange of bath solution, followed by repetitive depolarizations to +40 mV (from a holding potential of −100 mV) at 0.2 Hz, to achieve a steady-state level of block, before recordings. Glass microelectrodes were made from thin-walled borosilicate glass (World Precision Instruments) with a tip resistance of 0.2–0.8 MΩ when filled with 3 M KCl. Current signals were acquired at 10 kHz sampling frequency and were low-pass filtered at 4 kHz (−3 dB, 8-pole Bessel filter).

### hERG channel expression in HEK cells

HEK-293 cells were cultured in Dulbecco’s modified Eagle’s medium supplemented with 10% FBS at 37°C with 5% CO_2_. WT or R56Q mutant hERG pcDNA was cotransfected with GFP in pcDNA using lipofectamine 3000 (Invitrogen), and after 14–16 h, cells were plated onto coverslips. Cells exhibiting green fluorescence were selected for whole-cell patch-clamp recording. Whole-cell patch-clamp recordings were performed using an Axon Instruments 200B amplifier and Digidata 1440 interface. Signals were acquired at 10 kHz sampling frequency and were low-pass filtered at 4 kHz. Cells were perfused (2 ml/min) with external solution containing (in mM) 140 NaCl, 4 KCl, 1.8 CaCl_2_, 1 MgCl_2_,10 glucose, and 10 HEPES, pH 7.4 with NaOH. Borosilicate glass capillaries (Sutter Instruments) were pulled using a P-97 puller (Sutter Instruments) to create patch electrodes, which were filled with internal solution containing (in mM) 130 KCl, 1 MgCl_2_, 1 CaCl_2_, 10 EGTA, 10 HEPES, and 5 Mg^2+^ATP, pH 7.2 with KOH. Patch electrodes had a tip resistance of 3.7–4.5 MΩ. Series resistance was compensated 60–70%, with no leak subtraction, using the amplifier circuitry. Bath temperature was monitored and maintained at 37°C using a TC-344B Warner Instruments temperature controller unit with bath chamber thermistor, a heated platform, and inline perfusion heater. External bath solution contained (in mM) 140 NaCl, 4 KCl, 1.8 CaCl_2_, 1 MgCl_2_, 10 glucose, and 10 HEPES, pH 7.4 with NaOH. Drugs were dissolved in DMSO and diluted to the desired concentration in external solution. Application of RPR260243 and dofetilide at different concentrations was achieved by 5 min perfusion of the compound. Upon whole-cell patch formation, recordings were made once the hERG tail current amplitude during a voltage step to −65 mV applied after a 2-s depolarizing step to +20 mV was stable (holding potential was −80 mV; frequency of voltage steps was 0.2 Hz).

### Voltage protocols and data analysis

Data acquisition and analysis were performed using pClamp 10.2 (Axon Instruments) and SigmaPlot (Systat Software) software. Activation was measured during a voltage clamp protocol with physiologically relevant step durations that stepped the membrane potential from −100 to +60 mV in 10-mV increments for 250 ms (holding potential, −100 mV) before a 750-ms repolarizing step to −110 mV (inter-sweep was set to the minimum time allowing for full channel closure). Peak tail currents upon repolarization to −110 mV from each depolarizing voltage step were measured, normalized to the maximum peak tail current recorded, and used to plot G-V relationships. To measure deactivation kinetics, a 250-ms activating step to +40 mV (holding potential, −100 mV) was followed by 750-ms repolarizing steps to a range of voltages. The current decay observed during the 750-ms repolarizing step was fit with a two-term exponential function that afforded two time constants, τ_f_ and τ_s_, along with the amplitudes contributed by each component, A_f_ and A_s_, respectively. The percentage contribution of the fast component to overall current decay was calculated from the relative fraction, A_f_ / (A_f_ + A_s_). In this way, deactivation is assessed after channels are driven into open states, from which they rapidly inactivate and enter the relaxed state. Inactivation was measured using the rectification method described in Sanguinetti and Jurkiewicz (1990) from data recorded during the deactivation protocol outlined above. Maximal current amplitudes during the 750-ms repolarizing step were plotted against voltage to obtain the fully activated I-V relationship, and were plotted alongside the linear conductance observed at potentials ranging from −140 mV to −110 mV. The rectification factor R was calculated at each potential using the current plotted in the maximal I-V relationship, R = I_hERG_ / [G*n*(V_t_ – E_rev_)], where G is the maximal conductance of hERG, n is the activation variable at +40 mV, V_t_ is the test potential, and E_rev_ is the reversal potential. G-V_Act_ and inactivation–voltage relationships were fitted with a Boltzmann function, *y = *1/{1 + exp[(*V*_1/2_ − *V*)/k]}, where *y* is the normalized peak conductance, *V_1/_*_2_ is the half-activation or -inactivation potential, *V* is the variable (test) voltage, and *k* is the slope factor. Protective current was measured using a voltage protocol adapted from Perry et al. (2016). Cells were held at −100 mV before receiving a stylized action potential waveform. 90% repolarization was taken to be analogous to 90% of the action potential duration (APD_90_), and Δt from 90% repolarization is referred to as the coupling interval. Beginning at −80 ms from 90% repolarization, 40-ms depolarizing steps to 0 mV were delivered at 10–20-ms intervals up to a coupling interval of 380 ms. Protective currents recorded during premature depolarization steps to 0 mV were measured by extrapolating single exponential fits to the current back to t = 0. All protective current amplitudes were normalized to whole-cell conductance to permit comparison between constructs and conditions. Whole-cell conductance was calculated by fitting the region of linear conductance at strongly hyperpolarized potentials, e.g., between −110 and −140 mV, the slope of which is analogous to whole-cell conductance. Currents recorded in response to action potential waveforms were normalized to whole-cell conductance (at 21°C) or peak tail current recorded in the same cell (at 37°C) to permit comparisons.

For calibration and validation of the mathematical model, WT or R56Q membrane currents recorded at 21°C in *Xenopus *oocytes and at 37°C in HEK cells were used. We developed a series of protocols, including the previously described “staircase protocol” (Lei et al., 2019a), which enabled rapid collection of high-quality data from single cells (Fig. 8). We adopted an approach used in our previous studies (Beattie et al., 2018; Lei et al., 2019a, b), in which both calibration and validation data for the model were collected from the same cell using short, information-rich protocols. Specifically, we performed three sweeps of a protocol which consisted of the staircase protocol (Lei et al., 2019a; used for calibration), followed by shortened versions of traditional square activation and inactivation protocols, followed by a previously developed protocol comprising a complex series of action potentials (Beattie et al., 2018), ending with a repeat of the staircase protocol for quality control purposes.

In between the first and second sweeps of the protocol, we washed in the hERG activator RPR, and in between the second and third sweeps of the protocol, we washed in the hERG blocker dofetilide (which also blocks the effects of RPR; Kang et al., 2005). In this way, we were able to obtain “control” traces by subtracting the third sweep from the first, and “RPR” traces by subtracting the third sweep from the second. Repeating this for WT and R56Q hERG channels enabled us to obtain a set of cell-specific control and RPR traces for both. The dofetilide subtraction has the benefit of eliminating the effects of endogenous and leak currents (assuming the seal/leak does not change substantially during the recording). We used three of the highest quality recordings for each of WT and R56Q for parameter estimation.

### Statistical analysis

All data are expressed as mean ± SD (*n* = sample size). Statistical comparisons between means were conducted using two-tailed paired or unpaired Student's *t* test, or one-way ANOVA test as appropriate, with P < 0.05 taken as an indicator for statistical significance. P values are given, except when lower than 0.001, which is expressed as P < 0.001.

### Mathematical model

The mathematical model structure used was informed by the trade-off between parameter identifiability and goodness-of-fit (Whittaker et al., 2020). We have shown previously that the simple 8-parameter Hodgkin–Huxley-style hERG model of Beattie et al. (2013), as also used by Di Veroli et al. (2013) at physiological temperature, can reproduce complex hERG dynamics relevant to action potential behavior with a high degree of accuracy for WT hERG channel currents from manual and automated patch-clamp systems at a range of temperatures including physiological temperature (Beattie et al., 2018; Lei et al., 2019a, b). Nonetheless, as this study entailed investigation of the R56Q hERG mutation and the hERG activator RPR, both of which affect hERG deactivation, which does not appear to follow a single exponential time course (Vandenberg et al., 2012), we opted to extend the Beattie model to allow two time courses of deactivation. This structure has been used in previous investigations by Di Veroli et al. (2013) at ambient temperature and Li et al. (2016), although the exact number of parameters in the models depends on assumptions used about the symmetry of the activation/inactivation processes. In the model that we used, the current, *I*_Kr_, was modeled with a standard Ohmic expression given by

$$I_{Kr}=g_{Kr}\cdot O\cdot \left(V-E_{K}\right),$$ (1)

where *g*_Kr_ is the maximal conductance, *O* is the open probability in the model, *V* is the transmembrane voltage, and *E*_K_ is the K^+^ reversal potential, which was calculated according to

$$E_{K}=\;\frac{RT}{zF}\ln\left(\frac{{\left[K^{+}\right]}_{o}}{{\left[K^{+}\right]}_{i}}\right),\;$$ (2)

where *R* is the ideal gas constant, *T* is the temperature, *F* is the Faraday constant, *z* is the valency of the ions (1 for K^+^), and [K^+^]*_o_* and [K^+^]*_i_* are the extracellular and intracellular K^+^ concentrations, respectively. We assumed Hodgkin–Huxley-like inactivation with a single “h” gate, and a three-state Markov model for activation (states *C_2a_* − *C_1a_* − *O_a_*_,_). The system of equations is

$$\dot{C_{1a}}=\;b_{2}O_{a}+a_{1}C_{2a}-\left(a_{2}+\;b_{1}\right)C_{1a}\;,$$

$${\dot{O}}_{a}=a_{2}C_{1a}-b_{2}O_{a}\;,$$

$$C_{2a}=1-\left(O_{a}+C_{1a}\right)\;,$$

$$\dot{h}=\frac{\left(h_{\infty }-h\right)}{\tau_{h}}\;,$$

$$h_{\infty }=\frac{a_{h}}{\left(a_{h}+b_{h}\right)}\;,$$

$$\tau_{h}=\frac{1}{\left(a_{h}+b_{h}\right)}\;,$$

where the open probability in Eq. 1 is given by $O=O_{a}h.$ An equivalent fully Markov model is shown in Fig. 9 A with its states given by $IO=O_{a}\left(1-h\right),$ $C1=C_{1a}h,$ $IC1=C_{1a}\left(1-h\right),$ $C2=C_{2a}h,\;$ and $IC2=C_{2a}\left(1-h\right).$ The rates (*a*_1_, *a*_2_, *a_h_*, *b*_1_, *b*_2_, *b_h_*) are voltage-dependent functions described by 12 kinetic parameters, θ = {*p*_1_, …, *p*_12_}, as shown in Fig. 9 A. Simulations were run using Myokit (Clerx et al., 2016) and PINTS (Clerx et al., 2019a), with CVODE absolute and relative solver tolerances of 10^−8^. All codes and data required to reproduce our results are freely available at https://github.com/CardiacModelling/R56Q-modelling. To improve model predictions, simulations were run on three different cell datasets.

### Parameter inference

Briefly, maximum likelihood estimation was used to infer model parameters from the experimental data. As described previously (Clerx et al., 2019b), log-transforms were employed on the non–voltage-dependent parameters in the transition rates, i.e., *p*_1_, *p*_3_, *p*_5_, *p*_7_, *p*_9_, *p*_11_. We specified the following statistical model

$$I_{Kr}^{data}=I_{Kr}^{model}+\varepsilon ,$$

where $I_{Kr}^{model}$ is given by Eq. 1, and noise was assumed to arise from a normal distribution, ε~𝒩(0,σ^2^) (Lei et al., 2019a; Beattie et al., 2018). Under this scheme, the most likely parameter set is identical to that given by a least-sum-of-square-errors fit, so the log-likelihood of a given set of parameters for recording *i* is proportional to

$$L_{i}\infty -{\sum \left(I_{Kr,i}^{model}-I_{Kr,i}^{data}\right)}^{2},$$ (3)

where the sum is over time points in the current trace for the calibration protocol C (Fig. 8). Our parameter inference scheme was repeated independently for WT and R56Q cells. We used the following assumptions in the scheme: (1) A set of “control” kinetic parameters, **θ**_Control_, exists, which is shared across cells. (2) Addition of the hERG activator RPR alters the kinetics such that a second set of kinetic parameters **θ**_RPR_ is again shared across cells. (3) Maximal conductance is cell-specific and not altered by RPR. (4) Variability across cells is assumed to be due to an offset in the applied voltage command, which is likely a leading cause of kinetic variability in patch-clamp recordings (Lei et al., 2019a, 2020). In the model equations, we thus defined the voltage as *V* = *V*_cmd_ − *V*_off_, where *V*_cmd_ is the intended applied voltage command, and *V*_off_ was computed according to *V*_off_ = *E*_K,measured_ − *E*_K_, where *E*_K,measured_ was determined on a cell-specific basis from the voltage at which current reversed in experimental recordings, and *E*_K_ is the Nernst potential given by Eq. 2. These assumptions gave us a likelihood of the form

$$\begin{array}{l} L\left(\theta_{Control},\theta_{RPR},\left\{g_{Kr,1},\ldots ,g_{Kr,N}\right\}\right)\propto \overset{N}{\underset{i=1}{\sum }}\\ \left[L_{Control,i}\left(\theta_{Control},g_{Kr,i}\right)+L_{RPR,i}\left(\theta_{RPR},g_{Kr,i}\right)\right] \end{array}$$

where N is the number of cells, and L_Control,i_ and L_RPR,i_, still defined by Eq. 3, are the control and RPR log-likelihoods, respectively, for the *i*^th^ cell. Three cells (*n* = 3) were used in the parameter inference for each of the WT and R56Q, so there was a vector of 27 parameters in total for each {θ_Control_, θ_RPR_, g_Kr,i_}_i = 1,…,N_ (12 control kinetic parameters + 12 RPR kinetic parameters + 3 cell-specific conductances). We selected only the three cells with highest data quality as this approach demands very good quality measurements to be maintained across the RPR260243 and dofetilide addition to give accurate subtracted traces, but it is important to note that we make cell-specific predictions (for protocols V1–V3 as seen in Fig. 8), which we then test to gain confidence in the accuracy of the inferred kinetic parameters.

### Whole-cell human ventricular action potential modeling

The model of *I*_Kr_ was subsequently incorporated into the O’Hara–Rudy dynamic (ORd) model of the human ventricular action potential (O’Hara et al., 2011) for investigations of whole-cell behavior. The maximal conductance, *G*_Kr_, was set to 0.038 A/F to give approximately the same APD at 1 Hz pacing frequency as that of the original ORd model. To investigate inducibility of action potentials during late repolarization, we applied rapid pacing at 2.5 Hz in order to reveal differences in protective current (due to differences in the amount that hERG deactivates during successive pulses), and then applied a short, suprathreshold stimulus 305 ms following the previous stimulus.

## Results

### Pharmacological slowing of deactivation kinetics rescues attenuated protective currents in response to premature depolarizations

**Figure 1.**
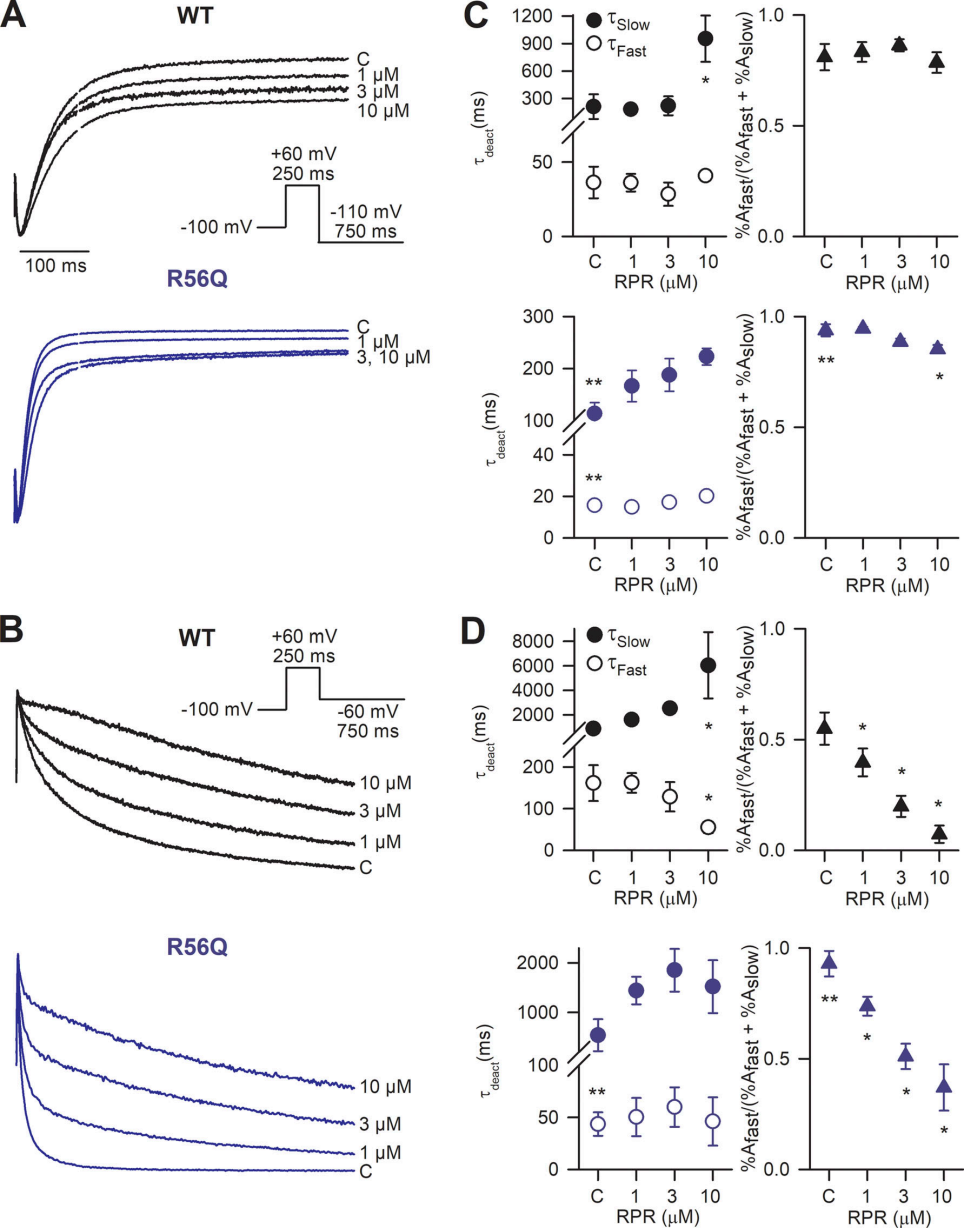
**RPR260243 (RPR) slows deactivation kinetics in WT and R56Q LQTS2-associated mutant channels in a concentration-dependent manner. (A and B)** Typical current traces recorded in response to the voltage protocol shown at 21°C from *Xenopus* oocytes (A), and at 37°C from whole-cell patch-clamp of HEK cells (B) during control conditions (C) and in response to application of RPR260243 at the indicated concentration. Recordings from WT channels are shown in black, and from R56Q mutant channels in blue. **(C and D)** Plots of mean deactivation time constants (left) and their relative amplitude (right) derived from double exponential fits of current decay during deactivation at 21°C (C; *n* = 5 for WT, *n* = 6 for R56Q) and 37°C (D; *n* = 5). Relative amplitudes are represented as the fast phase amplitude relative to the amplitude of total current decay (see Materials and methods, as well as Table 1). *, significant difference from control value; **, significant difference from WT control value.

**Figure 2.**
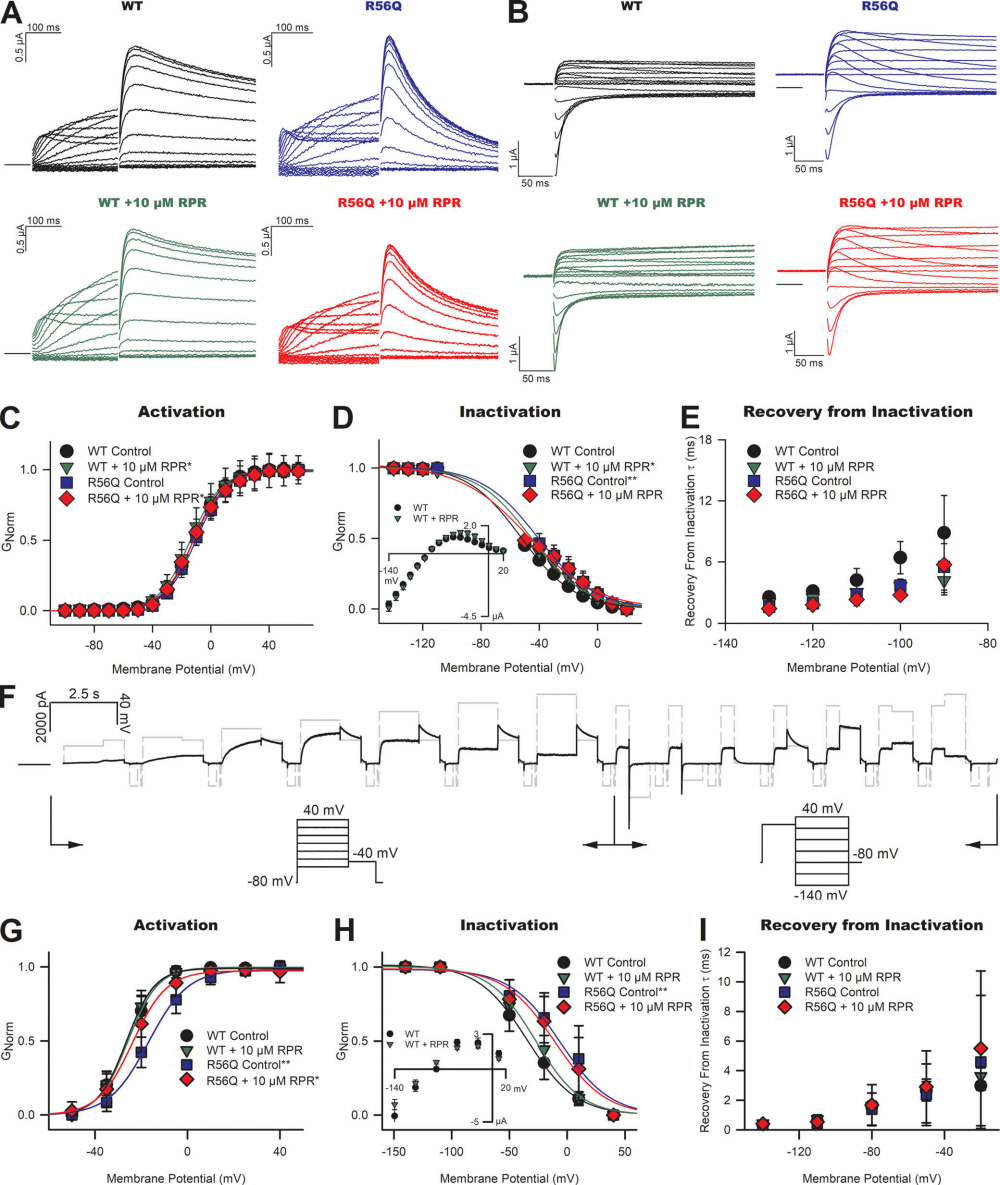
**Effects of RPR260243 (RPR) on biophysical properties of WT and R56Q mutant hERG channels. (A and B)** Typical current traces recorded at 21°C in response to activation (A) and deactivation/inactivation (B) voltage protocols of physiological duration from WT (left) and R56Q mutant (right) channels, with control recordings shown (upper), and recordings from the same oocyte in the presence of 10 µM RPR260243 (lower). **(C)** Plots of mean G-V relationships describing activation of WT and R56Q mutant channels in the absence and presence of RPR260243 (*n* = 7 for WT, *n* = 8 for R56Q). **(D)** Plots of mean G-V relationships describing inactivation in WT and R56Q mutant channels in the absence and presence of RPR260243 (*n* = 5 for both WT and R56Q). Inactivation–voltage relationships were calculated using the rectification approach from fully activated current–voltage relationships (inset) as described in the Materials and methods section. **(E)** Plots of mean τ values of the time course of the recovery from inactivation in WT and R56Q mutant channels in the absence and presence of RPR260243 (*n* = 5 for both WT and R56Q). **(F)** Typical current trace recorded from WT channels in response to a shortened single sweep voltage waveform used to derive gating parameters at 37°C (voltage waveform shown in gray). This information-rich voltage waveform protocol enables measurement of the voltage-dependence of activation (left portion between arrows) and inactivation (right portion between arrows) from a single sweep, improving efficiency of data collection at 37°C. To measure activation, currents were measured in response to successive 1,500-ms depolarizing steps applied from −50 mV to +40 mV in 15-mV increments followed by a 800-ms step to −40 mV (holding potential, −80 mV; time at holding potential between successive voltage steps, 200 ms). To measure inactivation, currents were measured from successive 800-ms repolarizing steps applied from −140 mV to +40 mV in 30-mV increments following a 500-ms depolarizing step to +20 mV. **(G–I)** Plots of mean activation (G) and inactivation (fully activated current voltage relationship used for rectification approach shown in the inset; H) G-V relationships, and the mean τ values for the kinetics of recovery from inactivation (I) recorded at 37°C. *, significant difference from control value; **, significant difference from WT control value.

**Figure 3.**
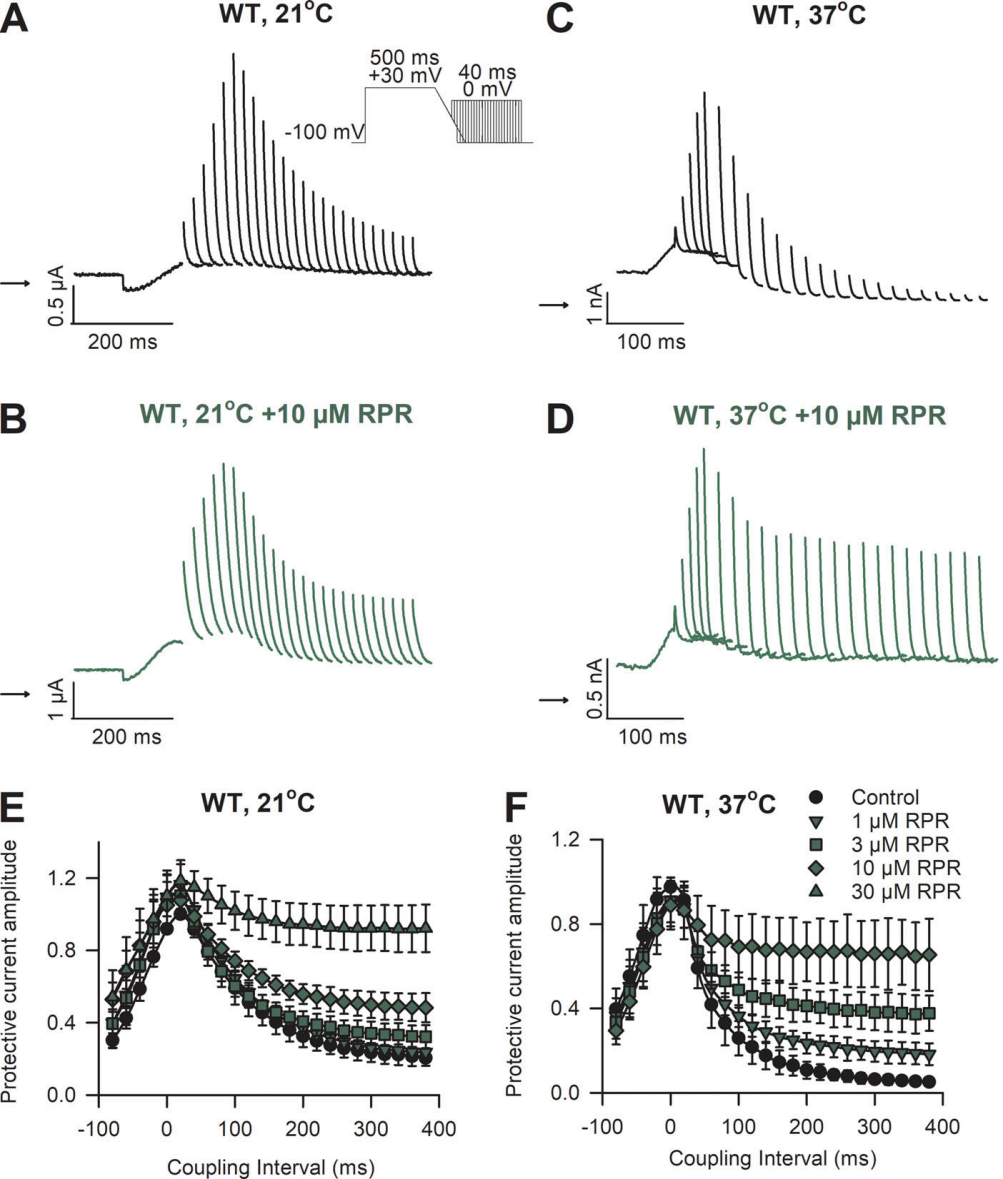
**hERG protective current is increased by RPR260243 (RPR) in a concentration-dependent manner. (A–D)** Typical current traces recorded from WT hERG channels in response to the premature stimulation protocol shown in the absence (black) and presence (green) of 10 µM RPR260243 at 21°C from *Xenopus* oocytes (A and B), and 37°C from HEK cells (C and D). **(E and F)** Mean peak current amplitudes in response to premature stimulations (protective currents) plotted against the coupling interval (Δt from 90% repolarization of the action potential-like waveform) recorded in the absence (black symbols) and presence (green symbols) of the indicated concentration of RPR260243 at 21°C (E; *n* = 5 for all data except *n* = 4 with 30 µM) and 37°C (F; *n* = 5).

Application of the hERG activator, RPR260243, caused a concentration-dependent slowing of deactivation kinetics in WT and R56Q mutant channels at both 21°C (in oocytes) and 37°C (in HEK cells). Fig. 1 shows that the R56Q mutation accelerated both fast and slow components of deactivation and increased the contribution of the fast component (see also Table 1). RPR260243 selectively delayed the slow component of deactivation in WT and R56Q mutant channels at both temperatures tested and increased its contribution at 37°C, but not at 21°C. Indeed, the typically fast deactivation kinetics in R56Q mutant channels became more WT-like in the presence of RPR260243. Fig. 2 characterizes the effects of RPR260243 on the biophysical gating properties of WT and R56Q mutant channels, besides deactivation. Step voltage waveforms were used to describe the voltage dependence and/or time dependence of activation and inactivation gating at 21°C (Fig. 2, A–E), while an information-rich shortened voltage “staircase protocol” waveform (Fig. 2 F; see also Materials and methods) was used to describe these parameters with improved efficiency at 37°C (Fig. 2, F–I). These data show that the effect of both the R56Q mutation and the action of RPR260243 were principally targeted toward deactivation, with a small additional effect on activation and inactivation, and no effect on recovery from inactivation (Fig. 2; see also Table 1). To assess the influence of manipulations of deactivation gating kinetics on hERG protective currents, we measured currents in response to premature stimulations (Fig. 3). Fig. 3, A–D, shows the effect of RPR260243 on WT channel currents at 21°C and 37°C during a voltage protocol (adapted from Perry et al., 2016) designed to mimic premature depolarizations arriving at different coupling intervals following a stylized action potential waveform. As previously observed, brief premature depolarizations (40 ms to 0 mV) applied during the refractory period following an action potential–like voltage waveform elicited robust transient repolarizing hERG currents, which have been described to be protective (Lu et al., 2001; Lu et al., 2003; Du et al., 2010; Du et al., 2013; McPate et al., 2009; Melgari et al., 2014; Perry et al., 2016). Peak hERG protective currents were normalized to maximal channel conductance and plotted against the coupling interval between 90% repolarization of the action potential–like waveform and the premature depolarization (Fig. 3, E and F; see Materials and methods). Similarly to previous descriptions (Lu et al., 2001; Lu et al., 2003; Du et al., 2010; Du et al., 2013; McPate et al., 2009; Melgari et al., 2014; Perry et al., 2016), premature stimulations produced robust transient repolarizing hERG protective currents in WT channels that peaked when the premature depolarization was applied at a coupling interval (Δt from 90% ramp repolarization) of 19 ± 5.2 ms (*n* = 15) at 21°C and −1.9 ± 8.9 ms (*n* = 5) at 37°C. Application of RPR260243 (Fig. 3, B and D) had little effect on the amplitude and timing of the peak hERG protective current recorded at any concentration tested (P = 0.16 at 21°C, and P = 0.56 at 37°C), but greatly enhanced hERG protective current amplitudes at longer coupling intervals in a concentration-dependent manner (Fig. 3, E and F). For example, the hERG protective current amplitude at a coupling interval of 100 ms was increased from 0.59 ± 0.09 that of the peak hERG protective current in control conditions to 0.74 ± 0.04 in the presence of 10 µM RPR260243 at 21°C (P = 0.017, *n* = 5; one-way ANOVA with Holm–Sidak post hoc test), while equivalent values at 37°C were 0.26 ± 0.09 and 0.69 ± 0.16 (P < 0.001, *n* = 5; one-way ANOVA with Holm–Sidak post hoc test). The presence of RPR260243 markedly increased hERG protective currents even at the longest coupling interval tested (375 ms), suggesting increased availability of channels during the diastolic period and the potential for enhanced rate-dependent accumulation of open channels with the activator (see below).

**Table 1.** Biophysical characteristics of WT and R56Q mutant channels in the absence and presence of RPR260243 (RPR) at 21°C and 37°C

		RPR	Activation		Deactivation (at −110 mV for 21°C, at −60 mV for 37°C)			Inactivation		Inactivation recovery
			V_1/2_ (mV)	k (mV)	τ_fast_ (ms)	τ_slow_ (ms)	% A_fast_	V_1/2_ (mV)	k (mV)	τ_-90mV_ (ms)
21°C	WT	Ctrl	−11.9 ± 2.8	10.3 ± 0.8	36.4 ± 10.6	166.8 ± 134	81 ± 5.9	−55.8 ± 6.1	−19.2 ± 0.9	8.8 ± 3.7
		1 µM	-	-	36.2 ± 5.9	182.8 ± 54	83 ± 4.4	-	-	-
		3 µM	-	-	28.5 ± 7.8	221.7 ± 102	86 ± 2.7	-	-	-
		10 µM	−14.1 ± 3.4^a^	10.6 ± 0.7	40.9 ± 3.1	953.7 ± 252^a^	79 ± 4.6	−44.0 ± 6.6^a^	−17.6 ± 0.8	4.2 ± 1.3
	R56Q	Ctrl	−10.5 ± 2.7	10.5 ± 0.3	15.7 ± 1.6^b^	114.0 ± 20.9^b^	94 ± 2.7^b^	−41.5 ± 8.5^b^	−19.6 ± 2.3	5.5 ± 2.3
		1 µM	-	-	14.9 ± 0.4	166.6 ± 30.1	95 ± 0.8	-	-	-
		3 µM	-	-	17.2 ± 1.8	188.0 ± 31.4	89 ± 1.5	-	-	-
		10 µM	−12.3 ± 3.2^a^	10.7 ± 0.6	20.1 ± 0.9	223.2 ± 16.1	85 ± 1.9^a^	−40.5 ± 8.3	−20.4 ± 1.6	6.3 ± 2.7
37°C	WT	Ctrl	−25.8 ± 3.1	6.2 ± 0.6	161 ± 43	866.8 ± 288	55 ± 7.3	−34.3 ± 10.4	−20.0 ± 0.5	1.5 ± 0.3
		1 µM	-	-	162 ± 24	1,595 ± 124	40 ± 6.3^a^	-	-	-
		3 µM	-	-	129 ± 35	2,526 ± 231	20 ± 4.8^a^	-	-	-
		10 µM	−26.5 ± 3.4	5.9 ± 0.7	54.9 ± 14^a^	6,029 ± 2,699^a^	7.2 ± 3.9^a^	−27.1 ± 6.8	−19.3 ± 1.7	1.6 ± 0.3
	R56Q	Ctrl	−15.8 ± 3.9^b^	7.9 ± 2.3	43.5 ± 5.1^b^	539.8 ± 322	93 ± 5.7^b^	−10.9 ± 16.9^b^	−19.9 ± 2.1	1.4 ± 1.1
		1 µM	-	-	50.2 ± 8.2	1,440 ± 276	74 ± 4.3^a^	-	-	-
		3 µM	-	-	59.8 ± 8.5	1,850 ± 432	51 ± 5.7^a^	-	-	-
		10 µM	−21.5 ± 6.0^a^	7.1 ± 1.6	46.0 ± 10	1,520 ± 533	37 ± 10.5^a^	−15.0 ± 14.7	−17.8 ± 4.4	1.7 ± 1.4

Ctrl, control.

a Significant difference compared to control (drug versus control).

b Significant difference compared to construct (R56Q versus WT).

**Figure 4.**
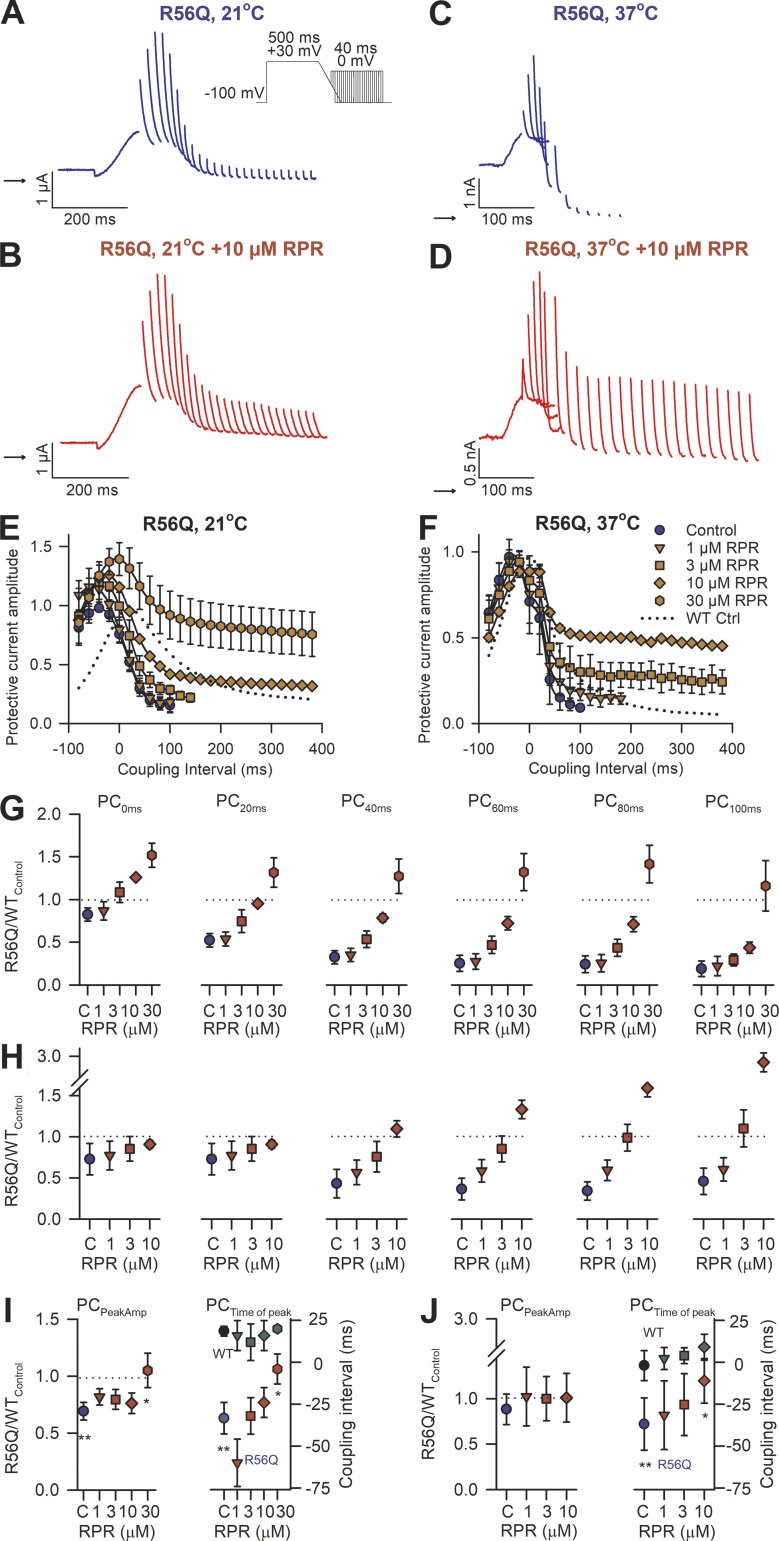
**RPR260243 (RPR) restores attenuated protective current in R56Q mutant channels. (A–D)** Typical current traces recorded from R56Q mutant channels in response to the premature stimulation protocol shown in the absence (blue) and presence (red) of 10 µM RPR260243 at 21°C from *Xenopus* oocytes (A and B) and 37°C from HEK cells (C and D). **(E and F)** Mean peak protective currents plotted against the coupling interval recorded in the absence (blue) and presence (red) of the indicated concentration of RPR260243 at 21°C (E; *n* = 5) and 37°C (F; *n* = 5). WT protective current amplitudes recorded under control conditions (absence of RPR260243) from Fig. 2 are also shown (dotted lines) for the purpose of comparison. **(G and H)** Plots of mean protective current at different coupling intervals in the refractory period (0–100 ms; PC_0–100ms_) in R56Q mutant channels as a fraction of that in WT channels in the absence (C, control; blue) and presence (red) of a range of RPR260243 concentrations at 21°C (G; *n* = 5) and 37°C (H; *n* = 5). Dashed black lines indicate WT values. **(I and J)** Left: Plots of mean peak protective current amplitude (PC_PeakAmp_) in R56Q mutant channels as a fraction of that in WT channels in the absence (C, control; blue) and presence (red) of a range of RPR260243 concentrations at 21°C (I; *n* = 5) and 37°C (J; *n* = 5). Dashed black lines indicate WT values. Right: Plots of mean coupling interval at which the peak protective current was observed (PC_Time of Peak_) in the absence and presence of RPR260243 in WT and R56Q mutant channels (*n* = 5). *, significant difference from control value; **, significant difference from WT control value.

The effect of RPR260243 application on hERG protective currents in response to premature stimulations in R56Q mutant channels is shown in Fig. 4. The R56Q mutation greatly reduced hERG protective currents in the refractory period compared with those in WT channels at both 21°C and 37°C (Fig. 4, A and C) as a result of accelerated deactivation gating. However, application of 10 µM RPR260243 increased R56Q hERG protective currents (Fig. 4, B and D). Fig. 4, E and F, plots R56Q hERG protective currents at different coupling intervals. In the absence of RPR260243, R56Q hERG protective currents at coupling intervals ≥0 ms were reduced compared with WT (dashed line). Application of RPR260243 resulted in a concentration-dependent increase in hERG protective currents passed by R56Q mutant channels in response to premature stimulations in the refractory period. Application of RPR260243 increased the R56Q hERG protective current such that the available repolarizing current was similar to, or even greater than, that in WT channels, depending on the drug concentration and the coupling interval. This can be seen more clearly in Fig. 4, G and H, which plots the hERG protective current recorded in R56Q channels as a fraction of WT channels at a number of coupling intervals and with increasing concentrations of RPR260243. In the absence of RPR260243 (Fig. 4, G and H, blue symbols), the R56Q mutation reduced hERG protective currents in response to premature depolarizations with the most prominent effect observed at a coupling interval of 100 ms (the longest coupling interval that produced a measurable protective current in R56Q mutant channels). At a coupling interval of 100 ms, the hERG protective current in R56Q mutant channels was 0.18 ± 0.09 (at 21°C) and 0.46 ± 0.16 (at 37°C) that of WT amplitude at 100 ms (Fig. 4, G and H). This R56Q hERG protective current at a coupling interval of 100 ms was 0.15 ± 0.07 (21°C, *n* = 5) and 0.09 ± 0.02 (37°C, *n* = 5) that of the peak protective current recorded in R56Q channels (Fig. 4, E and F). The peak hERG protective current in R56Q mutant channels also occurred at a shorter coupling interval than in WT channels (Fig. 4, I and J). The coupling interval in R56Q channels was −33.3 ± 16.3 ms compared with 18.7 ± 5.2 ms in WT channels at 21°C (*n* = 5, P < 0.001, *t* test) and −36.9 ± 13.9 ms compared with −1.9 ± 8.9 ms in WT channels at 37°C (*n* = 5, P < 0.001, *t* test). Additionally, the amplitude of the peak hERG protective current in R56Q mutant channels was reduced compared with that in WT channels at 21°C (P < 0.001), but not at 37°C (P = 0.271; Fig. 4, G and H).

Application of the highest concentration of RPR260243 tested increased R56Q mutant channel protective currents at a coupling interval of 100 ms 4.0 ± 0.9–fold at 21°C and 4.9 ± 1.1–fold at 37°C (Fig. 4, E–H). Approximate restoration of WT behavior at a coupling interval of 100 ms occurred in the presence of 30 µM RPR260243 at 21°C and 3 µM at 37°C, suggesting a possible temperature dependence to the effect size (see Discussion). Application of RPR260243 also increased the coupling interval at which the peak hERG protective current amplitude occurred in R56Q mutant channels at 21°C and at 37°C (Fig. 4, I and J). At the highest concentration of RPR260243 tested, the coupling interval at which the peak hERG protective current occurred in R56Q mutant channels was increased by 24.0 ± 16.8 ms at 21°C (*n* = 5, P = 0.033, paired *t* test) and 24.0 ± 8.9 ms at 37°C (*n* = 5, P = 0.004, paired *t* test), and became more WT-like. At 21°C, 30 µM RPR260243 also increased the peak hERG protective current in R56Q mutant channels, restoring the peak amplitude to WT-like control levels.

### RPR260243 restores attenuated early transient hERG channel currents during action potential waveforms with little effect on resurgent current

**Figure 5.**
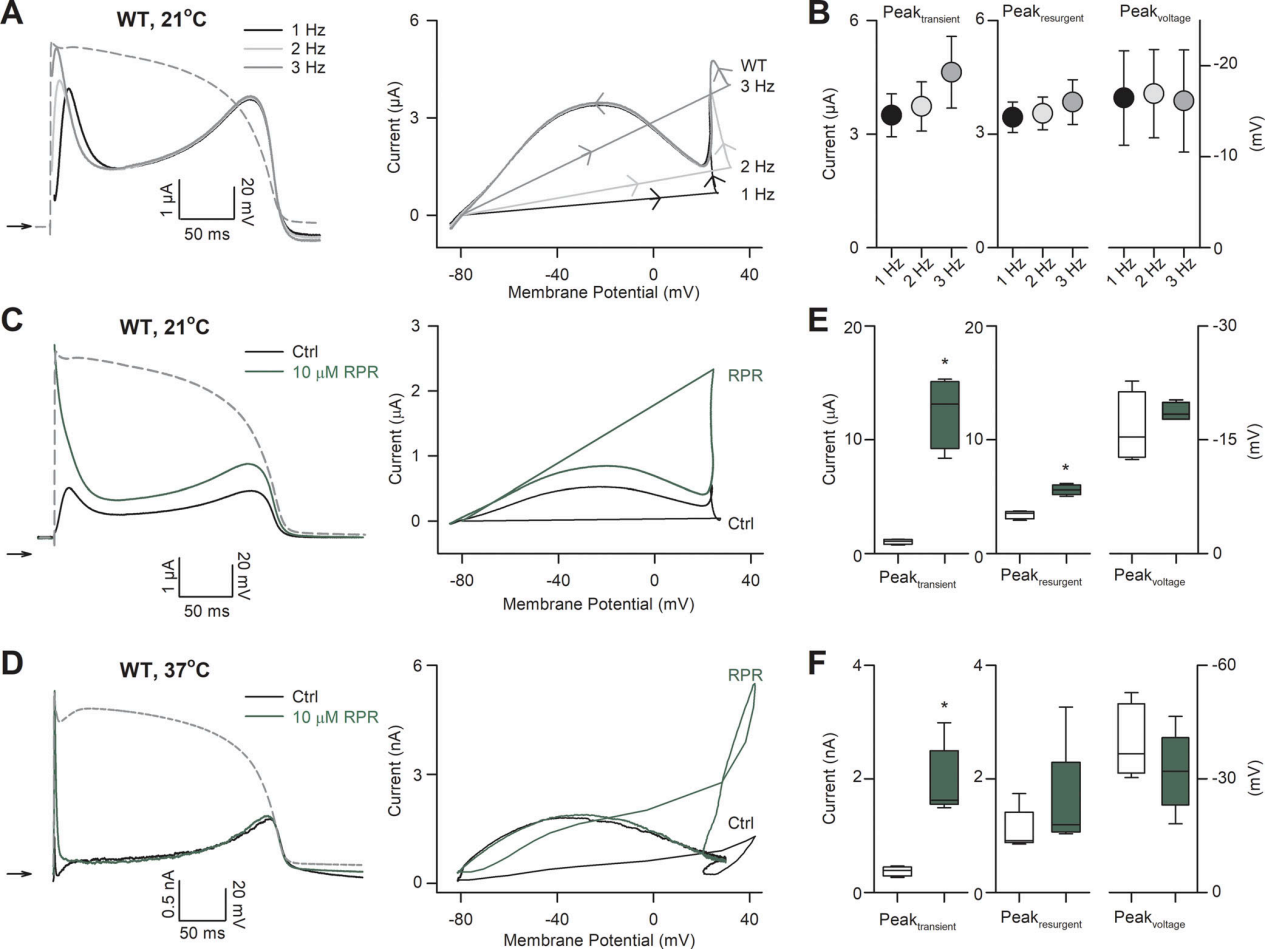
**RPR260243 (RPR) increases early transient hERG channel current during action potential voltage waveforms. (A)** Left: Typical current traces recorded from WT hERG channels at 21°C in response to the action potential voltage waveform shown by the dashed gray line applied at the indicated stimulation frequency. Right: Current–voltage relationships derived from WT hERG channel currents at the indicated stimulation frequency. **(B)** Plots of the mean peak early transient current (Peak_transient_, left), peak resurgent current (Peak_resurgent_, middle), and mean voltage at which the peak resurgent current occurred (Peak_voltage_, right) at the indicated stimulation frequency (*n* *= *4). **(C and D)** Typical current traces (left) and current-voltage relationships (right) recorded from WT hERG channels in response to the action potential voltage waveform shown (gray) applied at 1 Hz in the absence (black) and presence (green) of 10 µM RPR260243 at 21°C (C) and 37°C (D). **(E and F)** Plots of mean Peak_transient_, Peak_resurgent_, and Peak_voltage_ in the absence (control; open boxes) and presence (green boxes) of 10 µM RPR260243 at 21°C (E, *n* = 5) and 37°C (F, *n* = 5). Ctrl, control. *, significant difference from control value.

To investigate how the above findings influence repolarizing current during the action potential, we explored the effects of application of RPR260243 on hERG currents flowing during a simulated action potential voltage waveform at 21°C and 37°C (Fig. 5). Typical hERG current traces (e.g., in Fig. 5 A) describing the temporal development of hERG resurgent current during the action potential waveform were used to derive current–voltage relationships for quantitative evaluation of the effect of perturbations by mutation or activator compound. These relationships show that a robust transient hERG current is passed upon initial depolarization of the membrane followed by a slower hERG resurgent current that peaks in phase 3 of the action potential (Fig. 5 A). Voltage waveforms applied with increasing frequency resulted in an increase in the transient hERG current without appreciable effect on the hERG resurgent current (Fig. 5, A and B). This finding is consistent with the observation that the transient hERG current is conducted by open channels that failed to close during the previous diastolic period (Hua and Gilmour, 2004; Perry et al., 2016). Application of 10 µM RPR260243 increased WT hERG currents flowing during the action potential waveform when applied at 1 Hz at 21°C and 37°C (Fig. 5, C–F). The transient hERG current, whose amplitude was 1.05 ± 0.22 at 21°C and 0.36 ± 0.11 at 37°C relative to the peak of the hERG resurgent current, was dramatically increased 12.4 ± 4.6–fold at 21°C (*n* = 4, P = 0.007, paired *t* test) and 5.3 ± 1.3–fold at 37°C (*n* = 5, P = 0.002, paired *t* test) by 10 µM RPR260243. In contrast, the effect on the hERG resurgent current was more muted, with 10 µM RPR260243 causing a 1.6 ± 0.2–fold change at 21°C (*n* = 4, P < 0.001, paired *t* test) and a 1.4 ± 0.2–fold change at 37C° (*n* = 5, P = 0.035, paired *t* test). There was an equally modest change in the timing of the peak of the hERG resurgent current during the action potential waveform in the presence of RPR260243 (Fig. 5, E and F), which reached significance at 37°C (P = 0.046), but not at 21°C (P = 0.358).

**Figure 6.**
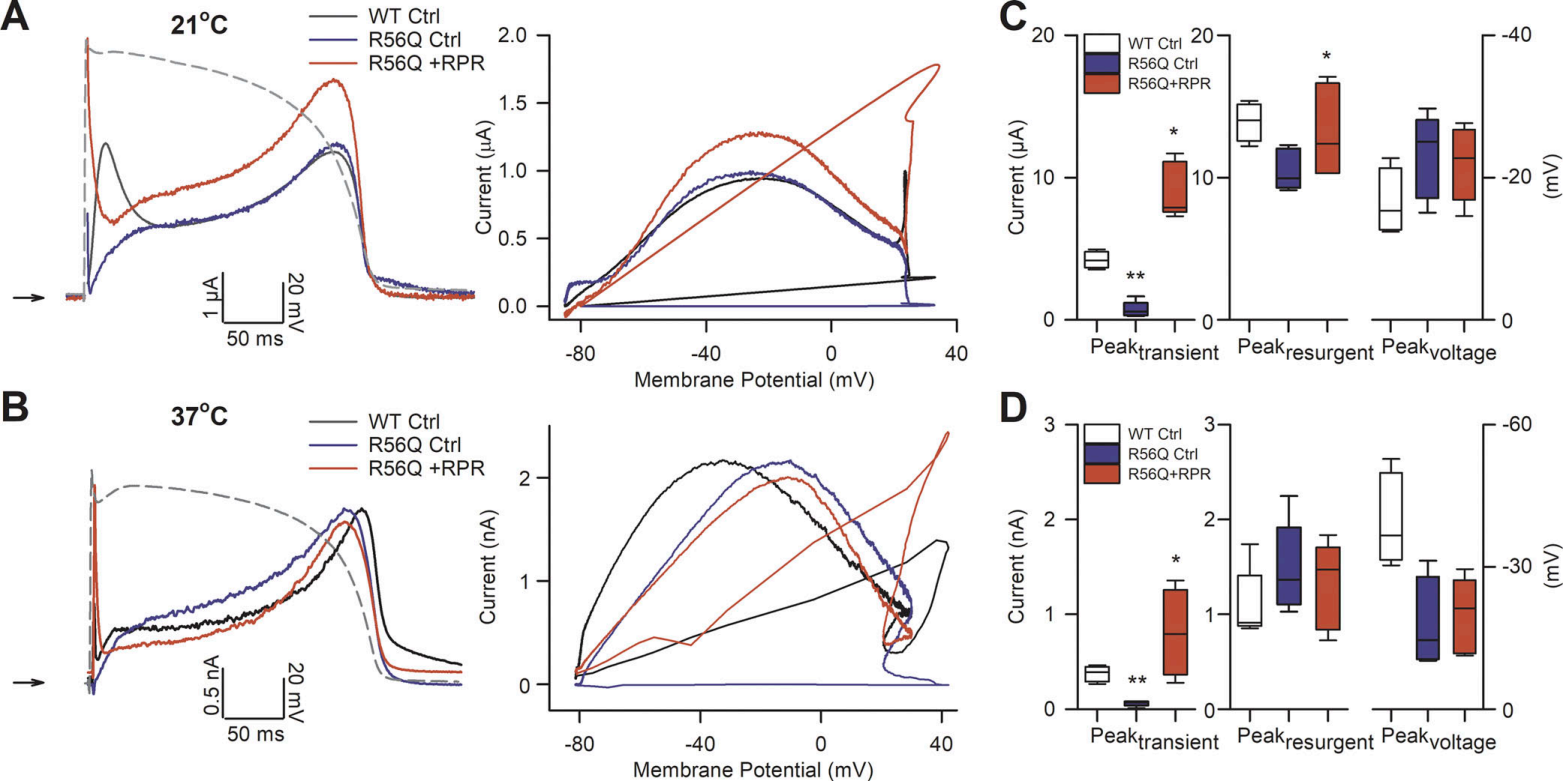
**RPR260243 (RPR) restores reduced early transient hERG current in R56Q mutant channels. (A and B)** Typical current traces (left) and current–voltage relationships (right) recorded from R56Q mutant channels in response to the action potential voltage waveform shown (gray) applied at 1 Hz in the absence (R56Q Ctrl; blue) and presence (R56Q +RPR; red) of 10 µM RPR260243 at 21°C (A) and 37°C (B). WT data from Fig. 4 are shown for the purposes of comparison (WT Ctrl; black). **(C and D)** Plots of mean peak early transient current (Peak_transient_, left), peak resurgent current (Peak_resurgent_, middle), and mean voltage at which the peak resurgent current occurred (Peak_voltage_, right) in R56Q mutant channels in the absence (control; blue boxes) and presence (red boxes) of 10 µM RPR260243 at 21°C (C, *n* = 5) and 37°C (D, *n* = 5). WT data from Fig. 4 are shown for the purposes of comparison (open boxes). *, significant difference from control value; **, significant difference from WT control value.

We next investigated the effect of RPR260243 application on repolarizing currents conducted during an action potential waveform in R56Q mutant channels at 21°C (Fig. 6 A) and 37°C (Fig. 6 B). Comparison of the peak amplitude of the hERG resurgent current, and the voltage at which the peak of the hERG resurgent current occurred during the action potential, revealed that the R56Q mutation had only small effects compared with WT at both 21°C (Fig. 6 C) and 37°C (Fig. 6 D). These observations are consistent with previous findings that the R56Q mutation has limited effects on hERG resurgent current during the action potential (Chen et al., 1999; Berecki et al., 2005; Gianulis and Trudeau, 2011). In contrast, the R56Q mutation dramatically reduced the amplitude of the transient hERG current at both temperatures. The transient hERG current was almost absent in R56Q mutant channels. Time-matched measurements of the transient hERG current indicated that they were 0.06 ± 0.04 at 21°C and 0.05 ± 0.02 at 37°C that of the peak hERG resurgent current. Application of 10 µM RPR260243 largely restored WT-like transient hERG current amplitude in R56Q mutant channels, increasing the repolarization 15.4 ± 8.3–fold at 21°C (*n* = 5, P < 0.001, paired *t* test) and 10.9 ± 5.8–fold at 37°C (*n* = 5, P = 0.032, paired *t* test). On the other hand, the application of RPR260243 had limited effects on the peak of the hERG resurgent current through R56Q mutant channels: the peak hERG resurgent current amplitude with RPR260243 was 1.25 ± 0.18 (at 21°C, P = 0.042) and 1.01 ± 0.29 (at 37°C, P = 0.889) that without the activator; also, the timing of the peak of the hERG resurgent current was not significantly altered (P = 0.280 at 21°C and P = 0.313 at 37°C).

**Figure 7.**
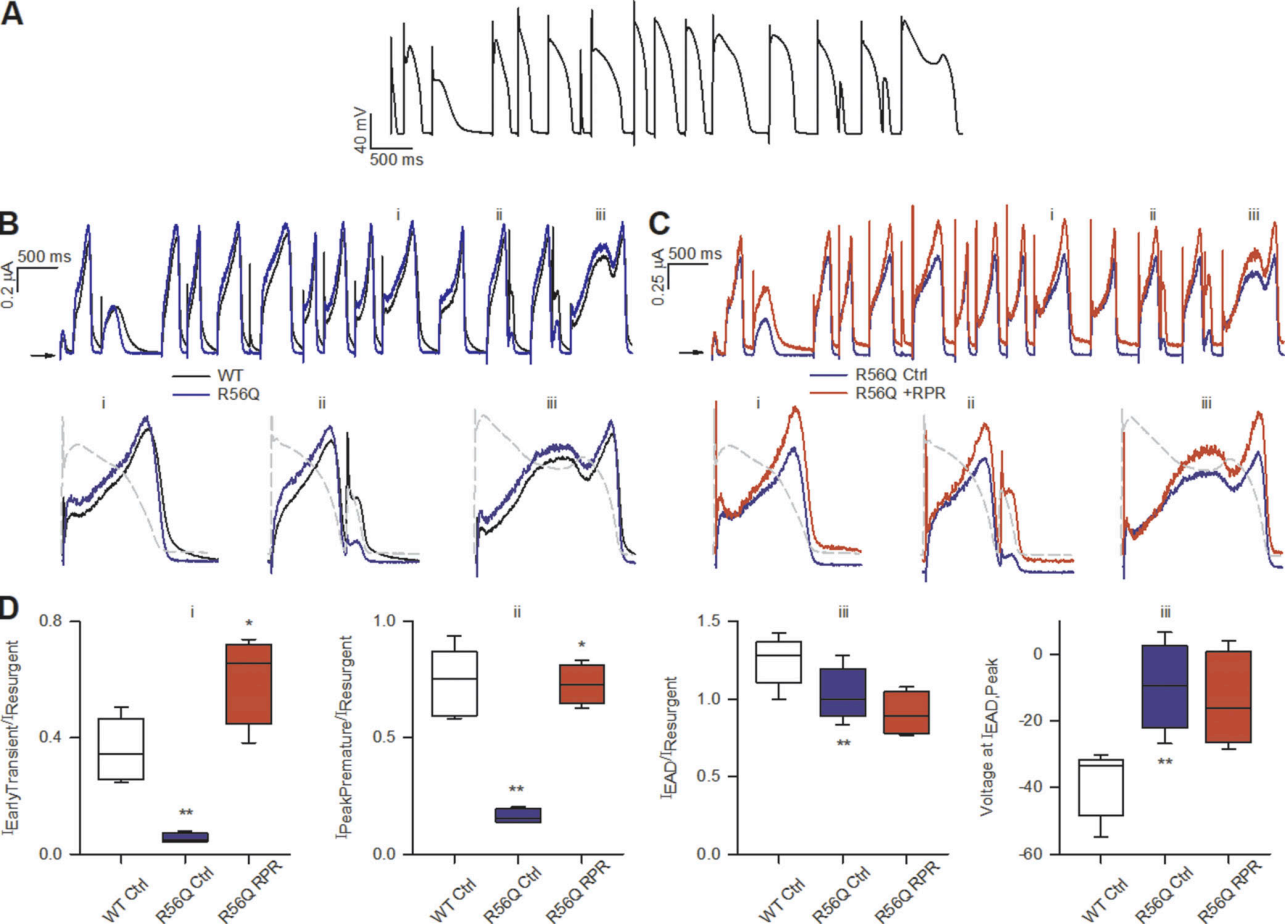
**RPR260243 (RPR) enhances attenuated protective repolarizing hERG current in R56Q mutant channels. (A)** Complex action potential voltage waveform train used to assess repolarizing current under a variety of stimulation scenarios. **(B)** Top: Typical current traces recorded from WT (black) and R56Q mutant (blue) channels at 37°C in response to the waveform in A. Bottom: Selected action potentials from the train (indicated by i–iii) highlight repolarizing current in WT and R56Q mutant channels passed in response to a ventricular-like action potential waveform (i, left), a premature depolarization arriving early in the refractory period (ii, middle), and an EAD (iii, right). **(C)** As in B, but with typical traces recorded from R56Q mutant channels in the absence (Ctrl, control; blue) and presence (+RPR; red) of 10 µM RPR260243. **(D)** Plots of mean repolarizing current parameters from i–iii in WT under control conditions (WT Ctrl; open boxes; *n* = 5) and R56Q mutant channels in the absence (R56Q Ctrl; blue boxes; *n* = 5) and presence of 10 µM RPR260243 (R56Q RPR; red boxes; *n* = 5). Plots show the amplitude of the early transient current relative to the peak resurgent current (i), the amplitude of the protective current in response to premature depolarization relative to the peak resurgent current (ii), the peak amplitude of repolarizing current during an EAD relative to the peak resurgent current (iii, left), and the voltage at which the peak repolarizing current during an EAD occurred (iii, right). *, significant difference from control value; **, significant difference from WT control value.

To explore these findings further, we applied a train of action potential waveforms with heterogeneous morphology and compared the current conducted by WT and R56Q mutant channels at 37°C (Fig. 7). This train of complex waveforms commands voltage changes that might be predicted from a range of cardiac tissue types and includes simulated afterdepolarizations (Beattie et al., 2018; Cooper et al., 2016). This enabled assessment of the effects of the mutation and drug on a range of action potential morphologies, rather than a single stylized ventricular action potential, to provide greater predictive insight. An overlay of typical currents elicited by this complex series of action potential waveforms in WT and R56Q mutant channels normalized to maximal channel conductance is shown in Fig. 7 B with hERG currents during selected action potentials expanded below (Fig. 7 B, i–iii). Consistent with our hypothesis, these typical overlays show only a small difference in the R56Q hERG resurgent current compared with WT. In contrast, there was a pronounced reduction in the transient hERG current caused by the mutation (Fig. 7 B, i). This can be seen clearly in Fig. 7 D, i, which plots the mean transient hERG current relative to the hERG resurgent current in WT and R56Q mutant channels. Moreover, the typical current traces in Fig. 7 B, ii, show that the R56Q mutation caused a robust reduction in the hERG current available in response to premature depolarizations arriving early in the refractory period. In these action potential complexes, WT channels produced robust current in response to premature depolarization. Plots of the mean peak current amplitude in response to premature depolarization (Fig. 7 D, ii) show that these currents were reduced 4.5-fold in R56Q mutant channels (*n* = 4, P < 0.001, one-way ANOVA with Holm–Sidak post hoc test), suggestive of decreased protection against the induction of triggered activity.

Fig. 7 C plots the overlay of typical current traces from R56Q mutant channels in the absence and presence of 10 µM RPR260243 with selected action potentials expanded in Fig. 7 C, i–iii. Application of RPR260243 restored the reduced transient hERG current in R56Q channels to WT-like levels (Fig. 7 C, i; and Fig. 7 D, i). Furthermore, RPR260243 also restored the peak repolarizing current passed in response to a premature depolarization (Fig. 7 C, ii), increasing the amplitude 4.6 ± 0.8–fold (Fig. 7 D, ii; *n* = 4, P < 0.001, one-way ANOVA with Holm–Sidak post hoc test). Interestingly, in action potential waveforms designed to mimic incidence of EADs (Fig. 7 B, iii), the peak amplitude of the repolarizing current passed by R56Q mutant channels during the EAD was decreased when compared with that in WT channels (P = 0.035), and the peak occurred at more depolarized potentials (P = 0.004), i.e., earlier in the action potential (Fig. 7 D, iii). Neither of these effects appeared to be greatly altered by the application of 10 µM RPR260243 (Fig. 7 C, iii; and Fig. 7 D, iii).

### Rapid characterization of R56Q kinetics using a short voltage protocol

Our findings suggest plausible mechanisms by which the R56Q mutation may predispose arrhythmia and highlight actions of the activator, RPR260243, which may have potential therapeutic benefit. In silico modeling enables consolidation of these complex effects with the ability to provide meaningful translational insight. However, the ability to predict how mutations and activator compounds influence cellular electrophysiology, and thus behavior at larger scales, depends critically on a faithful characterization of ion channel kinetics.

**Figure 8.**
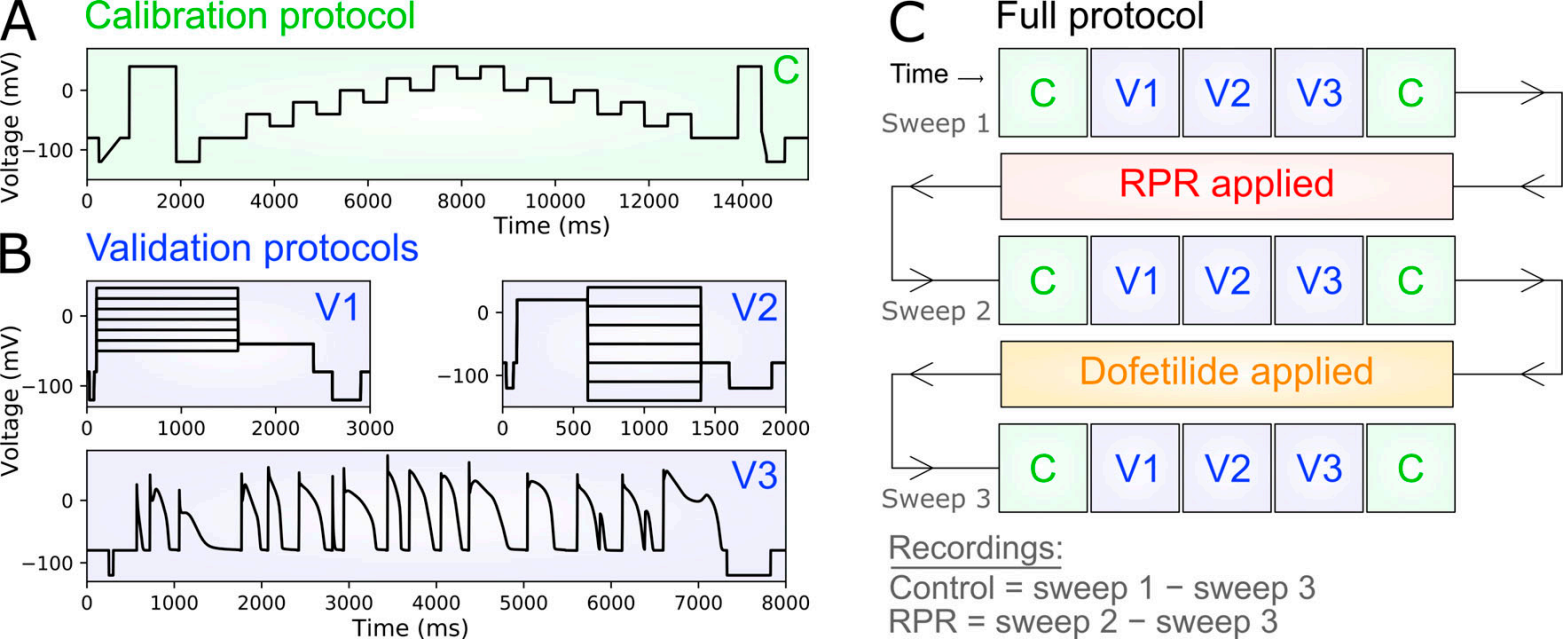
**Electrophysiological voltage waveform protocols used to characterize hERG channel ionic current kinetics. (A and B) **A novel electrophysiology protocol consisting of one calibration protocol (A) and three validation protocols used to build and test a model of hERG ion channel kinetics (B). **(C)** Sequence of protocols used in the full experiment, including control and RPR260243 (RPR) sweeps.

**Figure 9.**
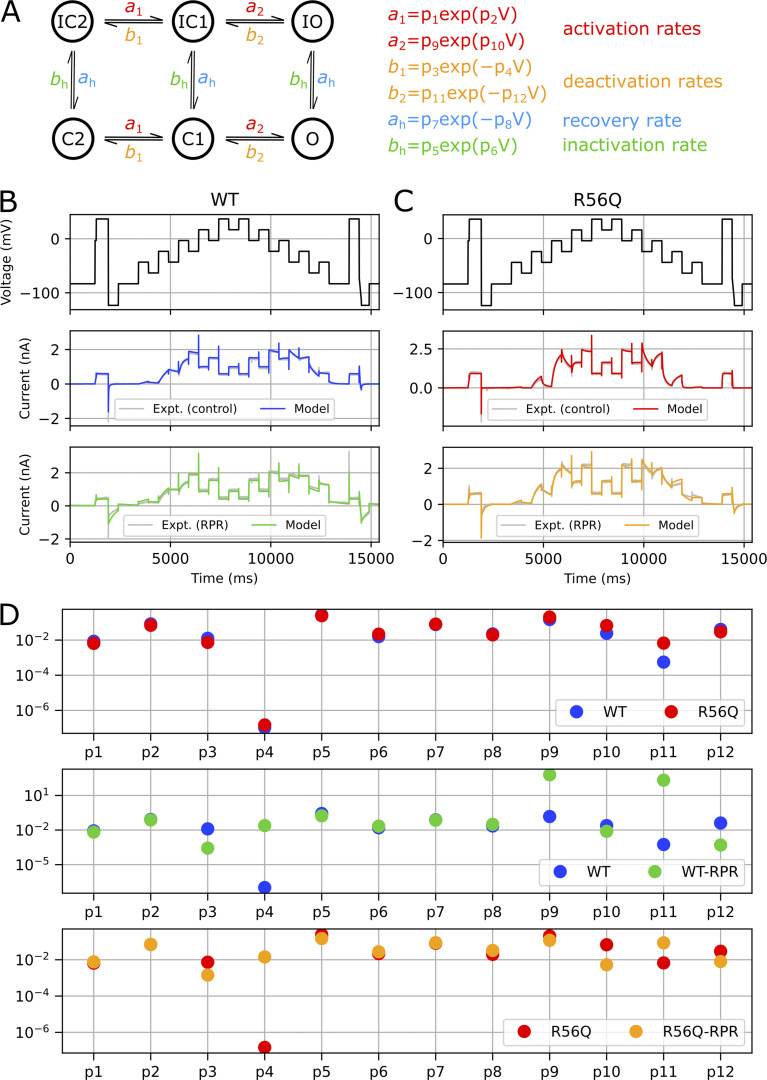
**Model structure, calibration, and parameters. (A–C)** The *I*_Kr_ model structure used. Control and RPR260243 (RPR) model fits to the calibration data from representative cells for WT (B) and R56Q (C). **(D)** Obtained kinetic parameters for (top) WT and R56Q, (middle) WT and WT-RPR, and (bottom) R56Q and R56Q-RPR models. Parameters *p*_1_, *p*_3_, *p*_5_, *p*_7_, *p*_9_, and *p*_11_ have units of ms^−1^, whereas parameters *p*_2_, *p*_4_, *p*_6_, *p*_8_, *p*_10_, *p*_12_ have units of mV^−1^. Expt., experiment.

**Figure 10.**
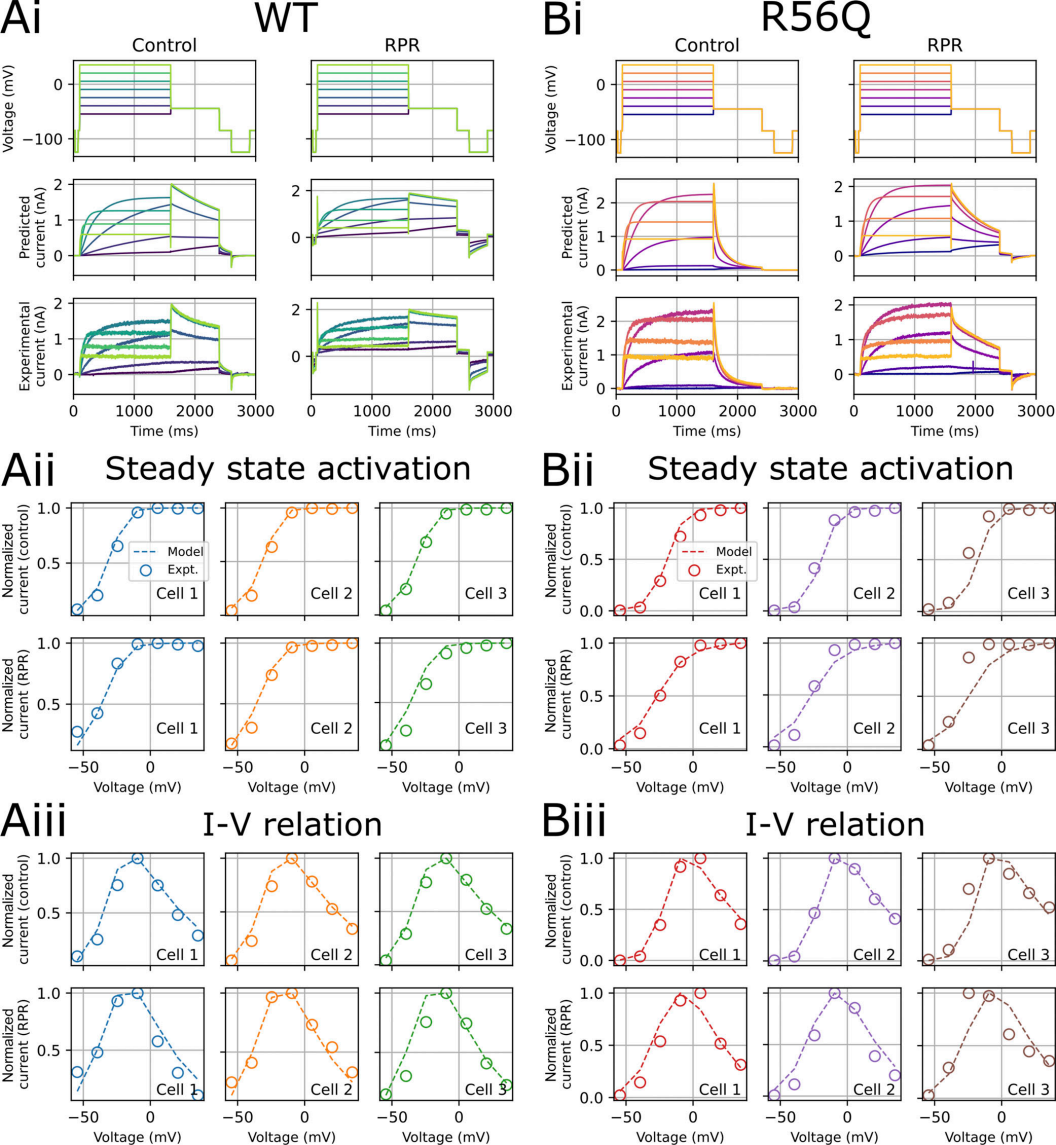
**Testing model predictions for activation properties. **A comparison of predicted currents from the mathematical model and experimental currents for the activation (V1) validation protocol for WT (A, i) and R56Q (B, i) control and RPR260243 (RPR) currents from a representative recording. Cell-specific predictions of the voltage dependence of activation (A, ii; and B, ii) and I-V relation (A, iii; and B, iii) for WT and R56Q channels, respectively. Expt., experiment.

**Figure 11.**
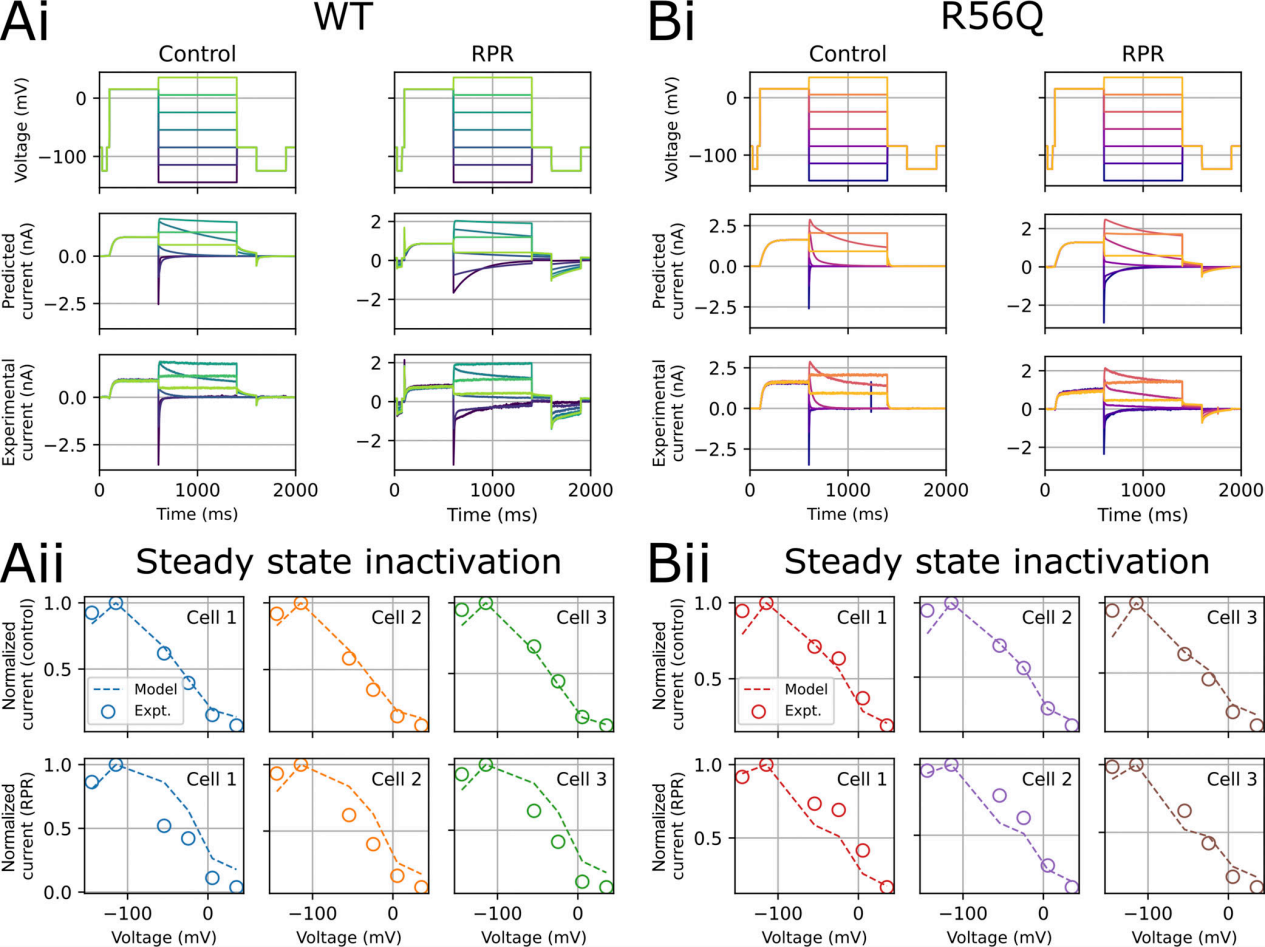
**Testing model predictions for inactivation properties.** A comparison of model predicted currents and experimental currents for the inactivation (V2) validation protocol for WT (A, i) and R56Q (B, i) control and RPR260243 (RPR) currents from a representative recording. Cell-specific predictions of the voltage-dependence of inactivation (A, ii; and B, ii) for WT and R56Q channels, respectively. Expt., experiment.

Fig. 8 shows the protocols used to calibrate and validate our modeling approach. We used information-rich protocols consisting of a staircase protocol for calibration, followed by validation protocols comprising step activation and inactivation voltage protocols, and a protocol applying a complex series of action potential–like voltage waveforms as used in our previous studies (Beattie et al., 2018; Lei et al., 2019a, b). Fig. 8 C demonstrates a typical experimental approach used to obtain dofetilide-sensitive hERG currents lacking endogenous and leak currents. The mathematical model scheme that we used is shown in Fig. 9 A, which is an extended form of a simple 8-parameter Hodgkin–Huxley style hERG model that was previously shown to reproduce complex hERG dynamics (Beattie et al., 2018; Lei et al., 2019a, b), such that it incorporates the known biphasic deactivation kinetics of hERG channels (Vandenberg et al., 2012). Representative model fits to the calibration data described in Fig. 8 are shown in Fig. 9, B and C. The model was capable of recapitulating the calibration data with a high degree of accuracy under control and RPR260243 conditions for both WT and R56Q channels. Kinetic parameters for the different cases are shown in Fig. 9 D (see also Table 2). Parameters for WT and R56Q control kinetics were highly similar, with the greatest difference being that *p*_11_ was an order of magnitude greater for R56Q than for WT. This parameter is responsible for the rate of deactivation along the O → C1 pathway, which is consistent with the accelerated deactivation in R56Q channels described above. Meanwhile, the *p*_4_ parameter for both WT and R56Q control kinetics was very small, which is consistent with the C1 → C2 transition being voltage-independent (Wang et al., 1997). Addition of RPR260243 affected the parameters in a more significant way, albeit in a manner that was consistent for both WT and R56Q. The main effect was to confer voltage dependence onto the C1 → C2 pathway by increasing *p*_4_. While RPR260243 greatly slowed the slow component of hERG deactivation, it actually appeared to increase the fast time course of deactivation, and thus did not reverse the increase in *p*_11_ associated with R56Q. Figs. 10 and 11 show calibrated model predictions of activation, deactivation, and inactivation gating behavior in WT and R56Q channels in the absence and presence of RPR260243 alongside experimental data. Fig. 10 shows predicted currents during step depolarizations in each condition (Fig. 10 A, i; and Fig. 10 B, i), and predicted steady-state activation (Fig. 10 A, ii; and Fig. 10 B, ii) and I-V relations (Fig. 10 A, iii; and Fig. 10 B, iii). Fig. 11 shows predicted currents (Fig. 11 A, i; and Fig. 11 B, i) and steady-state relationships (Fig. 11 A, ii; and Fig. 11 B, ii) describing inactivation gating. The predictions shown in Figs. 10 and 11 highlight that models calibrated using only the staircase protocol were able to predict activation and inactivation experimental data from the same cell with a high degree of accuracy. Moreover, the accelerated tail current decay associated with R56Q was reproduced with the control kinetics parameters. Addition of RPR260243 also slowed deactivation for both WT and R56Q. Excellent concordance between model and experiment was also seen when it came to cell-specific biomarkers corresponding to the activation and inactivation processes, such as the voltage dependence of activation and inactivation, and the I-V relation (Figs. 10 and 11).

**Table 2.** A summary of inferred parameter values from the mathematical *I*_Kr_ model

		WT	WT + RPR	R56Q	R56Q + RPR
*p_1_*		8.53E-03	6.67E-03	6.49E-03	7.66E-03
*p_2_*		8.32E-02	7.52E-02	6.97E-02	6.99E-02
*p_3_*		1.26E-02	2.71E-04	7.35E-03	1.44E-03
*p_4_*		1.04E-07	2.44E-02	1.49E-07	1.43E-02
*p_5_*		2.70E-01	1.72E-01	2.44E-01	1.48E-01
*p_6_*		1.58E-02	2.18E-02	2.18E-02	2.76E-02
*p_7_*		7.67E-02	7.12E-02	8.10E-02	8.74E-02
*p_8_*		2.25E-02	3.13E-02	1.98E-02	3.26E-02
*p_9_*		1.49E-01	6.21E+02	2.04E-01	1.19E-01
*p_10_*		2.43E-02	7.81E-03	6.74E-02	5.30E-03
*p_11_*		5.58E-04	2.10E+02	6.73E-03	8.60E-02
*p_12_*		4.07E-02	5.03E-04	2.94E-02	7.98E-03
	Cell 1	8.47E-02		7.96E-02	
*G*_Kr_	Cell 2	7.05E-02		1.03E-01	
	Cell 3	1.23E-01		4.33E-02	

Parameters *p*_1_, *p*_3_, *p*_5_, *p*_7_, *p*_9_, and *p*_11_ have units of ms^−1^, parameters *p*_2_, *p*_4_, *p*_6_, *p*_8_, *p*_10_, *p*_12_ have units of mV^−1^, and the maximal conductance parameters, *G*_Kr_, have units of μS.

**Figure 12.**
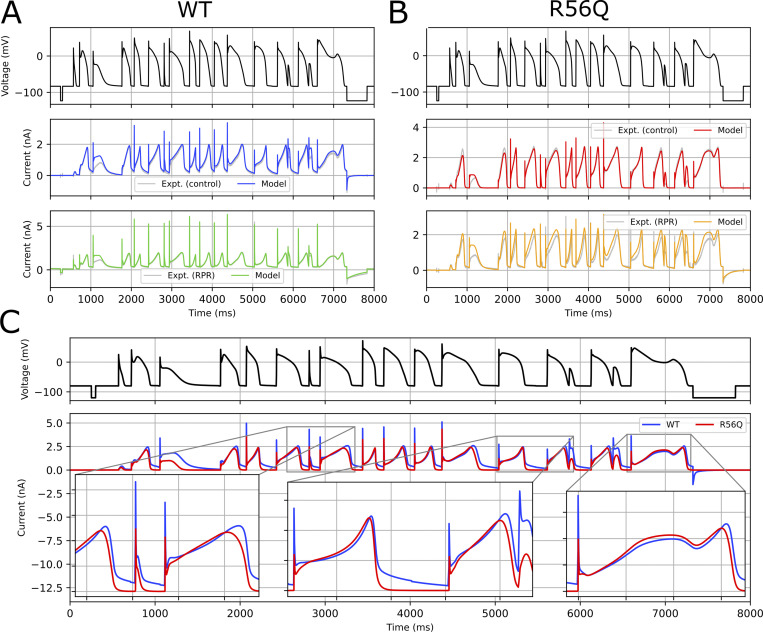
**Testing model predictions for currents during a complex action potential waveform voltage protocol.** **(A and B)** A comparison of experimental and model predicted currents from the complex action potential waveform (V3) validation protocol for WT (A) and R56Q (B) control and RPR260243 (RPR) currents from a representative cell. **(C)** A comparison of model currents for WT and R56Q control currents with maximal conductances (*g_Kr_*) fixed to be equal. Expt., experiment.

The calibrated models were also able to predict the complex action potential validation (V3) protocol with a high degree of accuracy in WT and R56Q channels in the absence and presence of RPR260243, as seen in Fig. 12, A and B, which shows an overlay of predicted and experimental currents in each condition. Fig. 12 C compares WT and R56Q kinetics directly by overlaying model output currents during the complex action potential voltage waveform protocol, with the model maximal conductances set to be equal. The expanded sections of predicted current tracings reveal that R56Q channels deactivated more quickly during the late phase of the action potential clamp, and crucially produced smaller protective currents in response to premature stimuli applied during late repolarization (middle expanded section). However, resurgent currents during the action potential remained similar in magnitude and profile with WT. This hints at a subtle electrophysiological phenotype associated with the R56Q mutation, which does not present under normal conditions.

**Figure 13.**
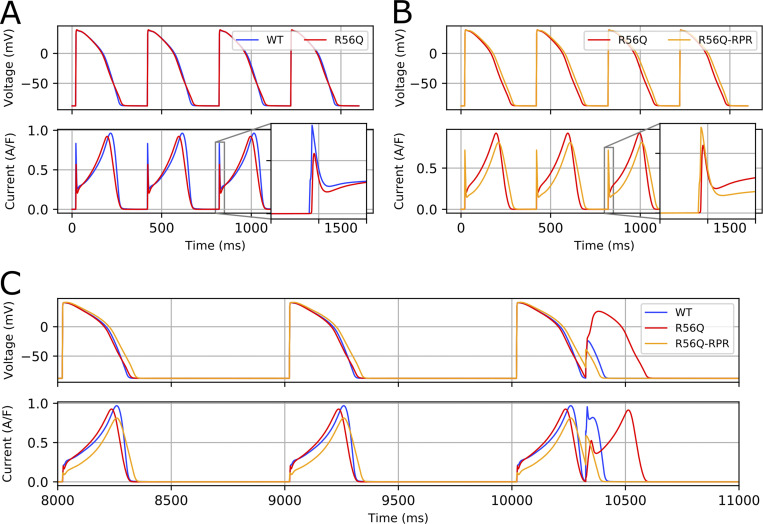
**Model predictions for effects of R56Q and RPR260243 on action potential characteristics. (A and B)** A comparison of action potentials and corresponding *I*_Kr_ for (A) WT and R56Q and (B) R56Q and R56Q-RPR at 2.5 Hz pacing in the ORd human ventricular model (endocardial variant). Insets show the protective current during action potential depolarization. **(C)** An example of the protective effect of RPR260243 (RPR), which prevents a premature stimulus from developing into a full action potential.

Fig. 13 shows the results of simulations in which the *I*_Kr_ current in the O’Hara–Rudy dynamic human ventricular action potential model (O’Hara et al., 2011) was replaced with the newly developed model from this study (with conductance chosen so that WT control kinetics produced the same APD as the original model). Under 1 Hz pacing conditions, the protective current arising from each successive depolarization was similar for WT and R56Q (data not shown). However, Fig. 13 A shows that with more rapid pacing, the transient hERG current was diminished by R56Q compared with WT. Addition of RPR260243 in R56Q conditions partially reversed the reduction in transient hERG current (Fig. 13 B). This may have an anti-arrhythmic effect at higher beating rates. Fig. 13 C shows action potentials and hERG currents simulated in the ORd model in WT and R56Q mutant channels and the influence of delivery of a stimulus during the late repolarization phase of the action potential. The premature stimulus did not result in a full action potential under WT control conditions, because a large, protective hERG current developed. In contrast, the protective current developed under R56Q control conditions was much smaller, and permitted the development of a full action potential that is more likely to propagate in tissue. This delayed afterdepolarization-like waveform can be a driver of reentrant excitations. Addition of RPR260243 restored some of the hERG protective current, again preventing development of a full action potential.

## Discussion

The main findings of this study are that the slowed deactivation gating caused by application of the type I hERG channel activator, RPR260243, (1) selectively enhances hERG protective currents conducted in response to premature depolarizations with limited effects on hERG resurgent current during the action potential, (2) rescues attenuated hERG protective currents in fast deactivating LQTS2-associated hERG R56Q mutant channels, and (3) is predicted to enhance impaired protection against afterdepolarizations and triggered activity. In addition, this study demonstrates further the robustness of a recently developed in silico modeling approach to thoroughly characterize and predict channel behavior in R56Q mutant channels at physiological temperature. This combined approach highlights a novel potential therapeutic opportunity for hERG channel activator compounds that slow deactivation gating, and provides a plausible mechanism for the pathogenicity and rescue of function in mutant hERG channels in which deactivation is accelerated, such as R56Q.

### RPR260243 selectively increases refractoriness and protection against premature depolarization

One of the main findings of this study is that by slowing channel deactivation kinetics, RPR260243 enhances hERG protective currents passed in the early refractory period in response to premature depolarizations. The effect of RPR260243 on hERG resurgent current during the action potential, by contrast, was significantly more muted. Slowed channel deactivation enables hERG channels to populate open conducting states and pass instantaneous robust repolarizing current in response to premature depolarizations (Miller, 1996; Lu et al., 2001; Perry et al., 2016). Such residual repolarizing current has been described as hERG protective current, because it is anticipated that it will increase refractoriness of cardiac tissue and reduce the incidence of afterdepolarizations (Lu et al., 2003; Du et al., 2010; Du et al., 2013; McPate et al., 2009; Melgari et al., 2014; Ng et al., 2014). In addition, increased hERG channel open probability during the diastolic interval contributes to transient hERG repolarizing current in response to the onset of subsequent action potential depolarization, and this transient hERG current becomes more pronounced at higher stimulation frequencies (Hua and Gilmour, 2004; Nuss et al., 1999; Royer et al., 2005). As such, early hERG transient current as a result of slow deactivation kinetics may also contribute to increased tissue refractoriness and reduced arrhythmogenicity (Hua and Gilmour, 2004). Our findings suggest that targeted manipulation of the kinetics of deactivation using a type I hERG activator selectively enhances hERG protective currents and the early transient hERG current.

We used RPR260243, the first hERG activator to be discovered (Kang et al., 2005), for these studies since its action is well-characterized to cause profound slowing of hERG channel deactivation with little or no effect on activation or inactivation gating at concentrations ≤10 µM, aside from a slowing of recovery from inactivation kinetics (Kang et al., 2005; Perry et al., 2007; Perry and Sanguinetti, 2008; see also Fig. 2). Over this concentration range, RPR260243 exerts little effect on APD in isolated mammalian hearts with no significant effect on human cardiac Na^+^ channels or KCNQ1 K^+^ channels, and only weak inhibition of L-type Ca^2+^ channels (10% at 10 µM; Kang et al., 2005). In the majority of cases, we avoided using higher concentrations of RPR260243, e.g., ≥30 µM, since at these concentrations the compound also induces a depolarizing shift in the voltage dependence of inactivation gating of hERG channels, more profoundly inhibits L-type Ca^2+^ channels, and abbreviates APD (Kang et al., 2005; Perry et al., 2007). In some instances, 30 µM RPR260243 was used in experiments at 21°C to determine concentration–response relations, since we found the effect of the compound to be less pronounced at this temperature than at 37°C. Our approach, however, was not designed to determine temperature dependence of RPR260243 effects, since two different expression systems were used. Use of the oocyte system at 21°C allowed comparison and validation of the in silico approach with previous studies conducted at room temperature (Beattie et al., 2018), while use of the HEK cell system at 37°C allowed channel kinetics to be studied at physiological temperature. Presentation of both data series provides opportunity for future comparisons to aid interpretation of studies conducted in oocytes at room temperature.

The binding site for RPR260243 has been mapped by mutagenesis studies and is located at the intracellular interface of the S4–S5 linker, S5, and S6 helices (Perry et al., 2007; Perry and Sanguinetti, 2008). Specific residues that influence the effect of RPR260243 on deactivation gating cluster to form a hydrophobic pocket behind S5 and S6 that, when occupied by RPR260243, is suggested to influence electromechanical coupling between the S4–S5 linker and the pore during closing (Perry et al., 2007). It is feasible that the effects of many variants that accelerate deactivation, e.g., mutations in the N- or C-terminal regions, would be restored by the action of RPR260243. However, evidence suggests that interaction between N- and C-terminal regions determines deactivation gating (Gianulis et al., 2013), and the effect of RPR260243 on deactivation appears to improve with unrestrained N- and C-terminal regions (Wu et al., 2015). These observations suggest that the restorative effects of RPR260243 may be influenced by the location of variants within these critical regions. For example, R56Q has been suggested to weaken N-terminal interactions with the channel (Gustina and Trudeau, 2009), and this may enhance the effectiveness of RPR260243. Future studies could examine the beneficial effects of RPR260243 in variants in different channel regions.

Measuring the effect of novel hERG activator compounds on hERG resurgent current during the action potential, and consequent evaluation of the capability to shorten APD, are a standard approach in the characterization of their therapeutic potential. However, the effect of hERG activator compounds on hERG protective currents conducted in response to premature depolarizations has not been well studied. Our evaluation of the effects of RPR260243 on hERG resurgent current in addition to hERG protective current and early transient hERG current suggests antiarrhythmic potential for RPR260243, enthusiasm for which has been muted given its limited ability to shorten the APD. We propose that the targeted action of RPR260243 could be expected to increase refractoriness and reduce afterdepolarization risk without shortening APD and predisposing arrhythmogenicity. Indeed, over-correction of hERG resurgent current during the action potential is a significant limiting concern in the development of hERG activator compounds that target inactivation gating, since they may predispose to short QT syndrome (Vandenberg et al., 2012). As such, our findings highlight a novel potential opportunity for hERG channel activator compounds that slow deactivation gating to provide targeted pairing of the mechanism of the activator with the mechanism of the variant dysfunction. Further testing is required to establish a similar targeted approach with variants that alter hERG channel inactivation gating paired with type 2 hERG activators, such as ICA-105574 or NS1643, which alter inactivation. In these cases, the net balance of the effects of the activator, e.g., NS1643, to prolong refractoriness, which would be anti-arrhythmic, and also abbreviate the APD, which might be pro-arrhythmic (Hansen et al., 2006), needs to be assessed in translational model systems as has been suggested previously (Perry et al., 2019).

### Pathogenicity of the R56Q mutation associated with reduced protection against premature depolarizations is rescued by RPR260243

Another key finding of this study is that selective pharmacological slowing of deactivation gating rescued function in an LQTS2-associated mutant channel that displays accelerated deactivation gating, R56Q (Chen et al., 1999; Perry et al., 2019). We elected to use the R56Q mutation to study because the variant results in a pronounced effect on deactivation gating (Chen et al., 1999). Based upon the observed accelerated deactivation kinetics caused by the R56Q mutation, the variant has been proposed to limit resurgent *I*_Kr_ delay repolarization and prolong the QT interval.

The R56Q variant was first described as being associated with LQTS by Chen et al. (1999), and the phenotype was characterized as predominantly affecting hERG channel deactivation gating. Since this initial description, the R56Q mutation has been used on numerous occasions to investigate the influence of modifications to hERG deactivation (Clancy and Rudy, 2001; Berecki et al., 2005; Gustina and Trudeau, 2009; Gianulis and Trudeau, 2011; Gonzalez et al., 2013; Liu and Trudeau, 2015; Foo et al., 2019). Several studies have attempted to better understand the pathogenicity of the variant, with some studies suggesting APD prolongation, particularly at higher rates (Clancy and Rudy, 2001; Berecki et al., 2005; Gonzalez et al., 2013; Liu and Trudeau, 2015), and other studies suggesting reduced surface expression (Foo et al., 2019). However, despite its initial association with LQTS, causation remains uncertain, and ClinVar assigns R56Q as a variant of uncertain significance. This picture highlights the challenges associated with risk stratification of hERG channel variants and the need for improved mechanistic insight to assign phenotypic risk with genotype. We believe this to be where our study provides particular insight. Our data suggest that variants, such as R56Q, are pathogenic and associated with sudden death despite minimal prolongation of the APD and QT_c_, because they reduce hERG protective currents providing a substrate for triggered activity and reentrant arrhythmia. As such, our data inform novel mechanisms that may guide improved risk stratification of known and novel hERG variants.

Previous studies assessing the R56Q variant suggested that the mutation does not greatly reduce, and may even increase, resurgent hERG currents during the action potential (Berecki et al., 2005; Liu and Trudeau, 2015). These effects can also be observed in the present study (Fig. 6), which may arise as a result of the right-shifted inactivation voltage dependence produced by the R56Q mutation (Fig. 2). Our whole-cell action potential modeling suggested that this greater “early” resurgent current would lead to a slightly depressed action potential plateau, causing a small prolongation overall when R56Q channels deactivate more rapidly during the repolarization phase of the action potential. It should be noted that we observed appreciably less APD prolongation in our R56Q model simulations compared with that observed previously (Berecki et al., 2005; Liu and Trudeau, 2015). In their dynamic clamp study, Berecki et al. (2005) applied a scaling factor to biological HEK R56Q hERG currents before injection into a dynamic action potential clamp simulated myocyte so as to normalize maximal peak current at ∼0 mV to that in WT. In our simulations, we assumed equal maximal conductance (the *g*_Kr_ term in Eq. 1) in WT or R56Q mutant channels, with differences in current magnitude arising from altered kinetics and thus different steady-state open probabilities. This approach led to milder APD prolongation than observed by Berecki et al. (2005). In simulations where we did apply the same scaling procedure (which corresponded to ∼6% reduction in R56Q maximal conductance, *g*_Kr_, based on peak current at 0 mV; data not shown), we observed greater APD prolongation, similar to that observed previously (Berecki et al., 2005). Our simulation data could not, however, recapitulate the very pronounced APD prolongation observed from R56Q current injection into rabbit myocytes (Berecki et al., 2005). To achieve similar prolongation of the APD in our simulations would require ∼75% reduction in hERG current (link to Cardiac Electrophysiology Web Lab experiment in Shannon–Bers rabbit ventricular cell model: http://bit.ly/ikr-block-shannon), which is not supported by our data. Interestingly, Liu and Trudeau (2015) also observed greater APD prolongation in human induced pluripotent stem cell–derived cardiomyocytes overexpressing R56Q mutant hERG channels than they expected from the effect of the mutation on current amplitude during voltage clamp experiments (Liu and Trudeau 2015). It is possible that this discrepancy is a result of an effect of the R56Q variant to impair trafficking. Some reports show that the R56Q mutation does not influence channel trafficking to the membrane (Chen et al., 1999; Gianulis and Trudeau, 2011), but more recent studies demonstrate a modest (∼20%) reduction of mutant channel surface expression due to increased internalization (Foo et al., 2019). These observations highlight the challenge of clinical risk stratification of individuals presenting with variants associated with inherited LQTS (Barsheshet et al., 2013; Giudicessi and Ackerman, 2013).

Our findings provide a plausible mechanism for pathogenicity in R56Q mutation carriers, as well as others harboring mutations that accelerate hERG channel deactivation. Our measurements from R56Q mutant channels show that the accelerated deactivation is associated with a profound reduction in hERG protective currents passed in response to premature depolarizations without marked effect on the hERG resurgent current flowing during the action potential. These findings predict a limited effect on APD and the QT interval, but increased risk of arrhythmogenicity as a result of reduced protective current against afterdepolarizations. We found that subsequent slowing of deactivation in R56Q mutant channels, by the application of RPR260243, produced a concentration-dependent restoration of the attenuated hERG protective currents. The extent to which protective currents were restored was dependent on the coupling interval, suggesting that a restorative effect over a broad range of coupling intervals in the refractory period might be achievable. The outcome of slowing of deactivation caused by RPR260243 resulted in limited changes to the hERG resurgent current during action potential voltage waveforms, but profound increase in hERG protective currents passed in response to premature depolarizations, as well as increased early transient hERG current passed upon initial action potential depolarization. These findings suggest a need to extend characterization of the effects of hERG variants associated with LQTS2 and sudden cardiac death beyond measurement of hERG resurgent current to include effects on hERG protective current (Perry et al., 2016). This enhanced approach to the functional screening of the pathogenicity of known and novel variants may contribute to improved risk stratification and understanding of the basis of concealed LQTS, in which individuals harbor a variant assumed to be pathogenic yet do not present with QT prolongation (Ackerman, 2008).

### Mathematical model of hERG kinetics predicts protection from arrhythmogenicity by RPR260243

To contextualize the findings of these studies, we used a mathematical modeling approach to generate simple predictive quantitative models of channel gating that could be well-constrained by experimental observations. The results of this approach suggest that the model can faithfully recapitulate channel behavior in a cell-specific manner. Adjustments in model parameters affecting deactivation and recovery from inactivation were sufficient to reproduce the changes in current observed under R56Q and RPR260243 conditions. Consistent with previous reports, the model predicted minimal effects on APD in R56Q due to the minimal effects of the mutation on the resurgent current. Following calibration using an information-rich short protocol (Beattie et al., 2018; Lei et al., 2019a, 2019b), the model was used to predict more complex channel activity during complex voltage waveforms and action potential trains. The model outputs were remarkably consistent with channel currents during these complex action potential trains in the case of WT and mutant channels, both under control conditions and in the presence of RPR260243. The model predicted decreased protective currents in response to premature stimuli during late repolarization in R56Q mutant channels, and partial rescue with RPR260243 application. Using this hERG gating model in the O’Hara–Rudy dynamic action potential model predicted reduced protective current with the R56Q mutation, particularly with more rapid pacing, compared with WT, which permitted development of a full action potential in response to premature depolarization in the mutant, but not WT. That R56Q hERG protective current was reduced at higher pacing rates is consistent with greater susceptibility of inherited LQTS2 carriers to cardiac events with elevated rates (Schwartz et al., 2001). The presence of RPR260243 partially restored the protective current, which suggests it may have a targeted an anti-arrhythmic effect at higher beating rates by resisting afterdepolarization waveforms, which can drive reentrant excitations.

The “rapid characterization” approach to modeling channel behavior used here provides faithful recapitulation of ion currents and steady-state parameter measurements, and accurately predicts behavior during complex waveform protocols. We found that such cell-specific predictive power can be sensitive to artifacts such as voltage offsets in patch clamp recordings that can cause the mathematical model to indicate changes in channel kinetics or conductance that may not be valid. As such, high-quality, low-leak, dofetilide-sensitive recordings with minimal rundown in each cell were required for high accuracy in the modeling process. An assumption of a constant maximal channel conductance (before and after RPR260243) was used to reduce the number of model parameters to infer; single channel measurements would offer one route to test this. We also identified rundown in channel activity during prolonged experiments as a factor that could influence the fitted model parameters and subsequent predictions. We did not correct for rundown in these studies, and future approaches could allow for a correction factor to be applied by incorporating controlled measures of rundown into the voltage waveform protocols. Another limitation in our approach is the weak inhibition of L-type Ca^2+^ channels (10% at 10 µM) by RPR260243 (Kang et al., 2005). While there were minimal effects on APD in guinea pig hearts (Kang et al., 2005), and our data suggest a targeted restoration of reduced protection in the refractory period in R56Q variant channels by RPR260243 that would not be expected to be influenced by weak inhibition of L-type channels, inhibition of I_Ca_ might be expected to affect action potential morphology, and this was not taken into account in our dynamic action potential simulations. However, simulations incorporating fractional I_Ca_ inhibition did not appreciably change the model outputs (data not shown), although further studies in translational models would help to address the overall balance of effects of RPR260243. Despite these limitations, this study demonstrates the robustness of this novel modeling approach to characterize and predict aberrant gating behavior and its rescue at physiological temperature. As such, our findings highlight the predictive power of this approach to risk-stratify the pathogenicity of hERG channel variants and the potential phenotypic gain in function following application of therapeutics.

Taken together, our findings suggest that modification of hERG channel deactivation, such as with RPR260243, presents a targeted approach to enhance refractoriness and reduce arrhythmogenicity without greatly shortening the APD. Such an approach may provide a lower risk alternative to the use of hERG activator compounds that target inactivation, which may over-correct loss of function. Furthermore, since RPR260243 enhances function in WT channels, its application may potentially prove useful in strategies to target haploinsufficiency, or non–hERG-related causes of LQTS, to improve protection against afterdepolarization.

## Data Availability

Data are available upon request. All codes and data required to reproduce our results are freely available at https://github.com/CardiacModelling/R56Q-modelling.
